# O-cyclic phytosphingosine-1-phosphate stimulates HIF1α-dependent glycolytic reprogramming to enhance the therapeutic potential of mesenchymal stem cells

**DOI:** 10.1038/s41419-019-1823-7

**Published:** 2019-08-05

**Authors:** Hyun Jik Lee, Young Hyun Jung, Gee Euhn Choi, Jun Sung Kim, Chang Woo Chae, Jae Ryong Lim, Seo Yihl Kim, Joo Eun Lee, Min Chul Park, Jee Hyeon Yoon, Myeong Jun Choi, Kye-Seong Kim, Ho Jae Han

**Affiliations:** 1 Department of Veterinary Physiology, College of Veterinary Medicine, Research Institute for Veterinary Science, and BK21 PLUS Program for Creative Veterinary Science Research, Seoul National University, Seoul, 08826 Republic of Korea; 2 Axcesobiopharma, 268 Hakuiro, Dongan-gu, Anyang, 14056 Republic of Korea; 3 Department of Biomedical Science, Graduate School of Biomedical Science and Engineering, Hanyang University, 222 Wangsimni-ro, Seongdong-gu, Seoul, 04763 Republic of Korea

**Keywords:** Transcription factors, Apoptosis, Lipid signalling, Protein translocation, Mesenchymal stem cells

## Abstract

O-cyclic phytosphingosine-1-phosphate (cP1P) is a novel chemically synthesized sphingosine metabolite derived from phytosphingosine-1-phosphate. Although structurally similar to sphingosine-1-phosphate (S1P), its biological properties in stem cells remain to be reported. We investigated the effect of cP1P on the therapeutic potential of mesenchymal stem cells (MSCs) and their regulatory mechanism. We found that, under hypoxia, cP1P suppressed MSC mitochondrial dysfunction and apoptosis. Metabolic data revealed that cP1P stimulated glycolysis via the upregulation of glycolysis-related genes. cP1P-induced hypoxia-inducible factor 1 alpha (HIF1α) plays a key role for MSC glycolytic reprogramming and transplantation efficacy. The intracellular calcium-dependent PKCα/mammalian target of the rapamycin (mTOR) signaling pathway triggered by cP1P regulated HIF1α translation via S6K1, which is critical for HIF1 activation. Furthermore, the cP1P-activated mTOR pathway induced bicaudal D homolog 1 expression, leading to HIF1α nuclear translocation. In conclusion, cP1P enhances the therapeutic potential of MSC through mTOR-dependent HIF1α translation and nuclear translocation.

## Introduction

Sphingosine metabolites are bioactive signaling lipids involved in many essential biological responses^1,2^. Several researchers have reported that sphingosine metabolites, such as sphingosine-1-phosphate (S1P) and phytosphingosine-1-phosphate (P1P), exert various physiological roles, including cell proliferation, differentiation, migration, immunomodulation, metabolism, and survival^3–6^. S1P is a representative zwitterionic lysophospholipid and is produced in yeast, plant, and mammalian tissues as a part of the sphingomyelin cycle^7^. The biological responses to extracellular S1P are based on the regulation of S1P receptors (S1PRs) belonging to G-protein coupled receptors^8^. Fingolimod and ponesimod are S1P receptor 1 (S1PR1) agonists, developed as immunomodulatory drugs to treat multiple sclerosis, psoriasis, and cancer^9^. Although previous findings indicate that sphingosine metabolites have great potential for cellular biological functions and therapeutic applications, their physicochemical nature and low yield cause their extraction and quantification to be challenging and expensive^10–12^. D-erythro-C18-Sphingosine-1-phospate is an S1P derived from the most common sphingoid base, D-erythro-C18-Sphingosine, in mammalian tissues^13^. The structure of S1P consists of a phosphate group at C1, an ammonium moiety at C2, and a hydroxyl group at C3 with a long-chain alkyl tail, essential for ligand recognition and specific receptor binding^14^. In addition, its D-erythro configuration is important for ligand binding affinity to S1PRs^14^. O-cyclic phytosphingosine-1-phosphate (cP1P) is a novel chemically synthesized sphingosine metabolite derived from P1P. The O-linked cyclication of phosphate group at C1 of P1P removes the hydroxyl group at C3 of P1P to form cP1P as a monohydric 18-carbon amino alcohols like S1P. Although cP1P’s regulatory effects on cell biology and function remain to be reported, determining the characteristic chemical structures of the O-linked cyclication of phosphate group and hydroxyl group will provide new biological properties to P1P through changes in the specificity and binding affinity to S1PRs.

Mesenchymal stem cell (MSC) transplantation has been considered a potential treatment for inflammatory, ischemic, and neurodegenerative diseases^15,16^. However, major limitations remain regarding the low transplantation efficacy caused by oxidative stress^17^. Recently, several investigators have tried to control stem cell metabolism, aiming to improve resistance to hypoxia by reducing oxidative stress^18,19^. Under hypoxia condition, oxygen availability is decreased by a metabolic switch from oxidative phosphorylation to glycolysis, thereby decreasing mitochondrial activity and ROS production^20^. Furthermore, previous studies have suggested a close relationship between intracellular metabolism and stem cell biology^20,21^. The maintenance of self-renewal, biological functions, and stem cell survival depends upon metabolic reprogramming, including glycolytic switch under hypoxia^22^. For example, metabolically adapting MSC through hypoxia preconditioning leads to increased bioactivities, such as proliferation, survival, and angiogenesis, which accelerate bone repair^23,24^. A previous in vivo study showed that hypoxia-preconditioned MSCs improve tissue regeneration and blood perfusion in hindlimb ischemia model^25^. Recent studies have also reported the physiological role of sphingosine metabolites, including S1P and ceramide, in hypoxia-induced glycolytic reprogramming and mitochondrial energetic metabolism^26–28^. In addition to the regulatory effect exerted by sphingosine metabolites on cell metabolism, S1P priming is an effective strategy to enhance the therapeutic efficacy of MSC treatment via cell trafficking, angiogenesis, and anti-inflammation^29^. However, the physiological role exerted by sphingosine metabolite-induced metabolic regulation on MSC’s therapeutic potential remains unclear.

Hypoxia-inducible factor 1 (HIF1) is a major transcription factor that induces metabolic reprogramming via upregulation of anaerobic glycolysis and downregulation of oxidative phosphorylation^20,30^. HIF1 is activated through strict control of its alpha subunit (HIF1α) by transcription, translation, posttranslational modification like prolyl- or asparaginyl hydroxylation and ubiquitination, and microtubule-associated nuclear transportation. However, the HIF1 beta subunit (HIF1β) is constitutively expressed and does not possess an oxygen-dependent degradation domain^31–34^. Therefore, it has been suggested that HIF1α plays an important role in the HIF1-regulated metabolism. In fact, stabilizing HIF1α through loss of the factor inhibiting HIF (FIH) or through the oxidative dimerization of the prolyl hydroxylase domain protein 2 (PHD2) facilitates metabolic adaptation to hypoxia^30,35^. Previous studies showed that HIF1α-overexpressed MSC exhibits high immunomodulatory effects and high resistance to hypoxia-induced apoptosis^36,37^. Taken together, the metabolic reprogramming of MSC by HIF1α regulation can be a promising strategy to improve MSC-based therapies. Furthermore, previous reports demonstrating that S1P acts as a HIF1α inducer suggest that cP1P, as a structural S1P analog, also has regulatory potential in the HIF1α-mediated glucose metabolism of MSCs^38,39^. Therefore, the present study investigated the regulatory effect of cP1P on the therapeutic potential of human umbilical cord blood-derived MSCs (UCB-MSCs) and its underlying mechanism.

## Materials & methods

### Materials

UCB-MSCs were kindly provided by Kangstem Biotech (Seoul, Korea), and its experimental use was approved by the Seoul National University Institutional Review Board (SNUIRB No E1707/002-003). UCB-MSCs were isolated and cultured as previously described^40^. UCB-MSCs’ characterization was performed by surface antigen profile verification and differentiation potential into tri-lineage, as previously reported^41,42^. Both P1P and cP1P were provided by Axceso (Seoul, Korea). cP1P was prepared by dissolving in 0.01 N NaOH solution. 0.01 N NaOH as a vehicle was treated to control group for cP1P treatment group. Sphingosine-1-phosphate (S1P, #S9666), 4’, 6-diamidino-2-phenylindol (DAPI, #23397 W), cycloheximide (#C4859), JTE013 (#J4080), VPC23019 (#857360 P), BAPTA-AM (#A1076), Rapamycin (#R0395), A23187 (#C9275) and PF4708671 (P20143) were purchased from Sigma-Aldrich (St. Louis, USA). EDTA (#PG205238) were purchased from Thermo Fisher Scientific (Waltham, MA, USA). Akt inhibitor (#124005) was purchased from Calbiochem (LaJolla, CA, USA). The mRNA primers for Na^+^/H^+^ exchanger isoform 1 (*NHE1*), solute carrier family 2 member 1 (*SLC2A1*)*,* *lactate dehydrogenase A* *(LDHA),* *phosphoinositide-dependent kinase 1 (PDK1), S1PR1, S1PR2, S1PR3, S1PR4, S1PR5* and *ACTB* were purchased from Bioneer (Daejeon, Korea). Small interfering RNAs (siRNAs) for *S1PR1, S1PR3, HIF1A, RPTOR, BICD1, PRKCA*, and nontargeting (NT) were purchased from Dharmacon (Lafayette, CO, USA). All chemicals were prepared at the time of use to avoid oxidation. All reagents were highly pure. The following antibodies were used: HIF1α (Abfrontier, Seoul, Korea, #YF-MA13455), prolyl hydroxylated HIF1α (Hyp402, Abcam, Cambridge, MA, USA, #ab72775), human nuclear antigen (HNA, Abcam, #NBP2-34525AF488), SMA (Abcam, #ab5694), CD31 (Novus Biologicals, # NB100-92205), vascular endothelial growth factor (VEGF, Santa Cruz Biotechnology, Dallas, TX, USA, #sc-7269), β-Actin (Santa Cruz Biotechnology, #sc-47778), E3 ligase von Hippel-Lindau (VHL, Santa Cruz Biotechnology, #sc-55506), epidermal growth factor (EGF, Santa Cruz Biotechnology, #sc-275), interleukin 6 (IL-6, Santa Cruz Biotechnology, #sc-7920), indoleamine 2, 3-dioxygenase 1 (IDO-1, Cusabio Biotech, Wuhan, China, #CSB-PA010996HA01HU), phospho pan protein kinase C (p-pan PKC, βII Ser660, Cell Signaling, Beverly, MA, USA, #9371), total PKC (Santa Cruz Biotechnology, #sc-10800), PKCα (Santa Cruz Biotechnology, #sc-208), p-PKCα (Ser657, Santa Cruz Biotechnology, #sc-12356), PKCδ (Santa Cruz Biotechnology, #sc-937), PKCε (Santa Cruz Biotechnology, #sc-214), pan-cadherin (Santa Cruz Biotechnology, #sc-10733), Lamin A/C (Santa Cruz Biotechnology, #sc-2068), β-Tubulin (Abfrontier, #LF-MA20056), α-Tubulin (Abfrontier, #LF-PA0146), p-Akt (Thr308, Santa Cruz Biotechnology, #sc-16646-R), p-Akt (Ser473, Santa Cruz Biotechnology, #sc-7985-R), Akt (Santa Cruz Biotechnology, #sc-8312), phospho mammalian target of rapamycin (p-mTOR, Ser2481, Cell Signaling, #2974), p-mTOR (Ser2448, Cell Signaling, #2971), mTOR (Cell Signaling, #2983), p-S6K1 (Thr389, Novus Biologicals, #NB600-1049), S6K1 (Abnova, Taipei, Taiwan, #H00006198-MD4), BICD1 (Novus Biologicals, #NBP1-78735), dynein intermediate chain (Dynein IC) (Santa Cruz Biotechnology, #sc-66866), Nrf2 (Cusabio Biotech, #CSB-PA003481), Ac-K68-MnSOD (Abcam, #ab137037), SOD2 (Cusabio Biotech, #CSB-PA003481), p-GSK3β (Ser9, Santa Cruz Biotechnology, #sc-11757), and GSK3β (Santa Cruz Biotechnology, #sc-9166).

### Cell culture of UCB-MSCs and SK-N-MCs

UCB-MSCs were cultured in a normal growth medium, containing α-minimum essential medium (α-MEM) provided by Thermo Fisher Scientific (Waltham, MA, USA), 1% penicillin–streptomycin (Gibco, Grand Island, NY, USA), and 10% fetal bovine serum (FBS, Hyclone, Logan, UT, USA). UCB-MSCs were grown in 6- and 12-well culture plates and 60 mm- and 100 mm-diameter cell-culture dishes (Thermo Fisher Scientific), kept in an incubator (37°C, CO_2_ 5%, air 95%). Twenty-four h prior to the experiments, the culture medium was replaced with serum free-α-MEM. After incubation, the cells were washed twice with phosphate buffered solution (PBS, Hyclone), then incubated in a medium supplemented with the indicated agents. The hypoxic environment was created by incubating UCB-MSCs in a hypoxia chamber (Billups-Rothenberg, Del Mar, CA, USA) with hypoxic gas (0.5% O_2_, 5% CO_2_, and 94.5% N_2_) at a 5 L/min flow rate for 15 min. The SK-N-MCs were delivered by the Korean Cell Line Bank (Seoul, Korea). The SK-N-MCs were cultured with high-glucose Dulbecco’s essential medium (DMEM; Hyclone), 10% FBS, and 1% penicillin–streptomycin (Gibco) in incubator (37°C, CO_2_ 5%, air 95%). The medium was replaced at 70% confluency of SK-N-MCs with DMEM with 1% FBS and incubated for 24 h. *RPTOR* knockout SK-N-MCs were established using the CRISPR/Cas9 system supplied by Dharmacon.

### Measurements of intracellular ROS, mitochondrial ROS, and mitochondrial membrane potential

The DCFDA (Thermo Fisher Scientific, #C6821), MitoSOX Red (Thermo Fisher Scientific, #M36008), and tetramethylrhodamine ethyl ester perchlorate (TMRE, Sigma-Aldrich, #87917) were used for measuring the intracellular ROS, mitochondrial ROS, and mitochondrial membrane potential, respectively. The detailed protocols were previously described^43^. The fluorescence intensity of DCFDA, MitoSOX and TMRE were measured at flow cytometer (CytoFlex; Beckman Coulter, Fullerton, CA, USA). Unstained cell data was presented as a fluorescence-minus-one (FMO) control of single fluorochrome staining. FMO control samples were applied to determine the positive part of flow cytometry data.

### Lactate dehydrogenase (LDH) cytotoxicity assay

Prior to the LDH concentration measurement, UCB-MSC cell concentration was optimized following the protocol provided by the LDH release assay (EZ-LDH, DoGenBio, Seoul, Korea, #DG-LDH500). UCB-MSCs (1 × 10^4^ cells/well) were seeded in a 96-well plate. The cells were grown at 90% confluency, and the media was replaced with serum free α-MEM. After treating for 72 h according to the experimental design, the plate was centrifuged at 600 × g for 5 min. The culture supernatant (10 μL) was collected and mixed with LDH reaction mixture (100 μL). After a 30-min incubation at room temperature, the optical density was measured at 450 nm using a microplate spectrophotometer (Epoch 2; BioTek, Winooski, VT, USA).

### Annexin V/PI apoptosis analysis-FACS

To evaluate UCB-MSC apoptosis, fluorescein isothiocyanate-conjugated annexinV (annexinV-FITC) and propidium iodide (PI)-double staining analyses were performed using an annexinV-FITC apoptosis detection kit (#556547, BD Bioscience, Franklin Lakes, NJ, USA) according to the supplier’s instructions. After treatment, cells (1 × 10^5^) were suspended in the binding buffer supplied with a commercial kit. Both annexinV-FITC (5 μL) and PI (5 μL) were added to the cell suspension solution, which was then incubated for 15 min at room temperature. UCB-MSC apoptosis was measured using flow cytometry (Beckman Coulter, Fullerton, CA, USA). Cells (3 × 10^3^) presenting similar side- and forward-scatter levels were measured using the flowing software2 (developed by Perttu Terho, Turku, Finland). AnnexinV-negative and PI-negative (Q3) cells were considered viable. AnnexinV-negative and PI-positive (Q1), annexinV-positive and PI-positive (Q2), and annexinV-positive and PI-negative (Q4) were considered as late apoptotic/necrotic, apoptotic, and early apoptotic cells, respectively. The percentage of total apoptotic cells was determined based on the following formula: Apoptotic cells = Q2 + Q4.

### WST-1 proliferation assay

UCB-MSCs proliferation and viability were determined using the WST-1 cell-viability assay kit (EZ-Cytox; Daeil Labservice, Seoul, Korea, #EZ-1000), according to the manufacturer’s instructions. Briefly, the UCB-MSCs cultured in 96-well plates were treated with cP1P, S1P, and P1P for 48 h. Cells were incubated in 10 μL of EZ-Cytox solution in 100 μL of medium for 30 min, at 37°C with 5% CO_2_. The absorbance was then measured using a microplate spectrophotometer (Epoch 2; Bio Tek Instruments, Winooski, VT, USA).

### Oris migration assay

Cells (1 × 10^4^) were plated onto each well of an Oris migration assay plate (Platypus Technologies, WI, USA, #CMACC1.101). The stoppers were gently eliminated and treated with cP1P, P1P, and S1P. The prepared plates were incubated for 24 h. Cells were stained with 5 μΜ of calcein AM (Thermo Fisher Scientific, #C1430) for 30 min. Cells migrating into the cell-free zone were detected using a microplate reader (Victor3; PerkinElmer, Norwalk, CT, USA) at excitation/emission = 485/535 nm.

### Tri-lineage differentiation of UCB-MSCs

For functional characterization, UCB-MSCs were plated in 12-well plates and differentiated into osteoblasts, chondrocytes, and adipocytes using specific differentiation media (StemPro Osteogenesis Differentiation Kit (#A1007201, Gibco), StemPro Chondrogenesis Differentiation Kit (#A1007101, Gibco), and StemPro Adipogenesis Differentiation Kit (#A1007001, Gibco). After 14–21 days, cells were fixed with 4% paraformaldehyde (PFA; Lugen Sci, Seoul, Korea, #LGB-1175) for 10 min and washed with PBS. To assess the osteogenic, chondrogenic, and adipogenic differentiation, cells were stained with alkaline phosphatase (ALP) staining, Safranin-O solution, and Oil Red O for 30–60 min, respectively and visualized using a microscope. To compare the adipocyte differentiation potential of MSCs, Oil Red O-stained cells were eluted in 100% isopropyl alcohol. Absorbance at 500 nm was measured by using spectrophotometer. Osteoblast differentiation level in UCB-MSCs was evaluated with Alkaline Phosphatase, Diethanolamine Detection kit (Sigma-Aldrich, #AP0100), according to the manufacturer’s instructions. UCB-MSCs were incubated in osteogenesis differentiation media (Gibco) or normal growth media for 14 days. Differentiated cells (1 × 10^5^) were lysed with 1% triton X-100 in PBS and centrifugated at 4°C, 15,000 rpm for 5 min. Supernatant was mixed with phosphatase substrate (p-Nitrophenyl Phosphate) in reaction buffer. ALP activity was measured with spectrophotometer at 405 nm, 37°C, for 20 min. ALP activity was calculated by comparison of the maximum linear rate (ΔA405 nm/min).

### RT2 glucose-metabolism PCR array

The glucose metabolism RT2 Profiler PCR Array (Qiagen, Valencia, CA, USA) was used to analyze glucose metabolism–related gene expression in cells treated with cP1P for 24 h according to the manufacturer’s instructions. In this array, a set of optimized primer assays allows mRNA transcript detection for 84 genes and five housekeeping genes in a rotor-gene style tube by Rotor-Gene Q (Qiagen, Hilden, Germany). PCR array data were analyzed using the GeneGlobe Data Analysis Center on Qiagen’s website. Upregulated glycolysis and downregulated TCA cycle genes with a fold change above 2 and p-value below 0.05 were selected.

### Hexokinase activity and lactate-production measurement

The hexokinase colorimetric (Biovision, Mountain View, CA, USA, #K789) and lactate colorimetric assay kits (Biovision, #K607) were used to measure UCB-MSCs hexokinase activity and lactate production. The assays were performed according to the manufacturer’s instructions. UCB-MSCs hexokinase activity and cellular lactate levels were measured with a microplate reader at 450 nm and 570 nm, respectively.

### Mitochondrial and glycolysis stress-test assays

The oxygen consumption rate (OCR) under mitochondrial stress-test assay and the extracellular acidification rate (ECAR) under glycolysis stress-test assay were performed using the Seahorse XF24 Extracellular Flux Analyzer (Agilent Technologies, Santa Clara, CA, USA). Mitochondrial and glycolysis stress-test assays were performed using an XF Cell Mito Stress Test Kit (Agilent Technologies, #103015-100) and XF Glycolysis Stress Test Kit (Agilent Technologies, #103020-100), respectively. The assays were performed according to the manufacturer’s instructions. The UCB-MSCs (1 × 10^4^ cells/well) were cultured in XF24 cell-culture microplates (Agilent Technologies, #100777-004). For mitochondrial stress-test assays, oligomycin (1 μM), carbonyl cyanide-4-(trifluoromethoxy)phenylhydrazone (FCCP, 0.5 μM) and antimycin A and rotenone mixture (0.5 μM) were treated to a cell-culture plate to determine the mitochondrial respiration, including basal respiration, maximal respiration, and spare respiratory capacity. For the glycolysis stress-test assay, D-glucose (10 mM), oligomycin (1 μM), and 2-deoxy-D-glucose (50 mM) were treated to a cell-culture plate to determine the glycolytic flux, including glycolysis, glycolytic capacity, and glycolytic reserve.

### Real-time quantitative PCR

UCB-MSCs were treated with cP1P or vehicle, after which the cells were washed twice with PBS twice and lysed with buffer-RL-added 50X DTT solution. The total RNA was extracted using an RNA extraction kit (Takara, Tokyo, Japan, #9767), according to the manufacturer’s instructions. Reverse-transcription PCR (RT-PCR) was conducted with 1 μg of total RNA with a Maxime RT premix kit (iNtRON, Sungnam, Korea, #25081). The cDNA was amplified using a Maxime PCR PreMix Kit (iNtRON, #25165) with a MyGenie 96 (Bioneer, Daejeon, Korea). The relative mRNA expression level of the target gene was analyzed using a Rotor-Gene 6000 device (Corbett Research, Cambridge, UK) with the TB Green Premix Ex Taq (TaKaRa, #RR420A). The specificity, efficiency, and fidelity of PCR primers for real-time quantitative PCR were validated by checking PCR products and melting curve analysis. Primer sequences are listed in Supplementary Table S1. The relative mRNA expression levels of the *SLC2A1*, *LDHA*, *PDK1*, and *NHE1* were analyzed using the delta-delta Ct method. The *18* *S rRNA* gene was used as the normalization reference gene.

### Intracellular pH measurement

Intracellular pH was measured by staining UCB-MSCs with BCECF-AM (Thermo Fisher Scientific, #B1150), an intracellular pH indicator. After treating cP1P for 24 h, cells were washed twice with PBS, after which the cells were incubated in 2 μM of BCECF-AM in PBS and kept at 37°C for 10 min. The cells were then rinsed with PBS. BCECF-AM fluorescence intensity was measured at excitation/emission = 485/535 nm with a microplate reader (Victor3).

### Small interfering RNA (siRNA) transfection

UCB-MSCs were incubated for 24 h with 25 nM of the indicated siRNAs and the transfection reagent TurboFect (Thermo Fisher Scientific, Waltham, MA, USA, #R0531), without antibiotics. The medium was changed to serum free α-MEM. The siRNAs sequences indicated in this study are described in Supplementary Table S2. NT siRNA was used as a control siRNA.

### Western blotting and subcellular fractionation

Protein concentrations were determined using bicinchoninic acid (BCA) protein-assay kits (Pierce, Rockford, IL, USA, #23225). Sample proteins were resolved by SDS-PAGE and transferred onto PVDF membranes, which were incubated overnight with the primary antibody at 4°C. The specific bands were visualized using the ChemiDoc XRS + System (Bio-Rad, Richmond, CA, USA). Subcellular fractionation was performed to isolate the cytosol, membrane, and nuclear proteins. Cells were cultured in 100 mm dishes and treated with the indicated reagents. An EzSubcell subcellular fractionation/extraction kit (Atto, Tokyo, Japan, #WSE-7421) was used to prepare the cytosolic, membrane, and nuclear fractionized samples. Cytosolic, membrane, and nuclear samples were prepared for western blot analysis according to the manufacturer’s instructions. Pan-cadherin and Lamin A/C were used as membrane and nuclear protein markers, respectively.

### HIF1 transcriptional activity measurement

To assess the transcriptional activity of HIF1, Cignal reporter assay system with HIF1-responsive dual firefly/renilla luciferase was taken from Qiagen (#CCS-007L). The HIF1 reporter activity was measured with a dual luciferase reporter assay system (Promega, Madison, WI, USA, #E1910). UCB-MSCs was treated with Cignal reporter construct (200 ng) and siRNA for *S1PR1*, *S1PR3*, and NT (25 nM) with Lipofectamine Stem transfection reagent (Thermo Fisher Scientific, #STEM0015) for 24 h, according to the manufacturer’s instructions. The dual luciferase reporter assay was conducted following the manufacturer’s instructions. The luciferase activities of firefly and renilla were measured using a luminometer (Victor3).

### Trypan blue exclusion cell viability assay

UCB-MSCs were transfected for 24 h with *HIF1A* siRNA or NT siRNA, after which the cells were treated with cP1P for 72 h. UCB-MSCs were washed twice with PBS and trypsinized into single cells. Cell suspension solution was centrifuged at 1,500 × g for 5 min. The cell pellet was suspended with PBS and stained with 0.4% trypan blue (Sigma-Aldrich, #T6146) in PBS (1:1 ratio). Cell viability was analyzed using Countess II automated cell counter (Thermo Fisher Scientific).

### Mouse skin flap model

All procedures using experimental animals were performed according to protocols approved by the Institutional Animal Care and Use Committee of the Seoul National University (SNU-181120-4). Eight-week-old male ICR mice were kept in a laboratory animal facility under a 12 h light/dark cycle and a temperature between 20 and 25°C. The mice were separated into five groups: vehicle-injected wild-type mice (group 1, *n* = 6); mice receiving UCB-MSCs pretreated with either NT siRNA alone (group 2, *n* = 6) or NT siRNA and 1 μM cP1P (group 3, *n* = 6); mice receiving UCB-MSCs pretreated with *HIF1A* siRNA and 1 μM cP1P (group 4, *n* = 6); mice receiving UCB-MSCs transfected with *HIF1A* siRNA (group 5, *n* = 6). After hair shaving, a 4 × 1 cm skin flap was made on the dorsal surface of the mouse. The whole process was performed under aseptic conditions, and a skin flap was left for 30 min maintaining body temperature and anesthesia. For treatment, 100 μL of a PBS solution containing the UCB-MSCs and the drug to be treated together was prepared and injected into the center of the skin flap, which was then returned to its original place and sutured. All the skin flap images were taken at the same distance from the subject (30 cm) with a digital camera system (D50; Nikon, Tokyo, Japan). All mice were sacrificed at postinjection day 12, after which 1.5 × 0.5 cm of the skin flap samples were collected. For H&E staining, half of each tissue sample was fixed with 10% formalin in PBS (Sigma-Aldrich) and embedded in paraffin with tissue processing. A 10-μm thick section was prepared. Another side of the tissue was embedded in OCT compound (Sakura Finetek, Tokyo, Japan, # HIO-0051). The histological analysis and scoring were performed in a blind fashion.

### Hematoxylin & Eosin staining

De-paraffinized slides were fixed with 4% PFA for 5 min, then stained with hematoxylin and eosin. Samples were washed three times with 70%, 95%, and 100% ethanol, after which they were incubated in xylene for 5 min. The cover slip was mounted with a mounting medium (EcoMount, Biocare Medical, Concord, CA, USA, #EM897L) and H&E stained slides were automatically scanned with Pannoramic SCAN (3DHISTECH Ltd. Budapest, Hungary). The skin flap’s necrotic areas were visually assessed using the ImageJ software (developed by Wayne Rasband, National Institutes of Health, Bethesda, MD, USA; http://rsb.info.nih.gov/ij/). Areas of the skin flap presenting dark color and scabs were considered necrotic. The necrotic portion of the skin flap was calculated using the following formula: Necrotic area in skin flap = necrotic area of flap area/area of total flap × 100. The tissue reepithelization of the skin flap was assessed according to a criteria described in Supplementary Table S3.

### Immunohistochemistry

Skin samples on slides were fixed for 20 min in an 80% acetone solution. Slides were washed in PBS and incubated for 30 min in 5% normal goat serum (Sigma-Aldrich, #566380). The samples were immunostained with HNA/DAPI, α-SAM/CD31/DAPI in PBS containing 0.2% Tween-20 (PBST) for 2 h, after which they were stained with Alexa Fluor 488 or 555-conjugated secondary antibodies, in PBST for 1 h. Fluorescence images of tissue samples were captured by Eclipse Ts2 fluorescence microscopy (Nikon, Tokyo, Japan) and analyzed with the MetaMorph software (Universal Imaging, West Chester, PA, USA).

### Immunocytochemistry

For immunocytochemistry, UCB-MSCs were fixed with 4% PFA for 10 min, then incubated in 0.5% Tween-20 for 10 min. Cells were incubated for 2 h with primary antibodies, in PBS containing 0.1% Tween-20 (PBST; 1:100 dilution), then washed three times with PBS. Cells were then incubated with Alexa Fluor 488 or 555-conjugated secondary antibodies in PBST (1:100 dilution) for 1 h. Immunofluorescence-stained samples were visualized using a super-resolution radial fluctuations (SRRF) imaging system (Andor Technology, Belfast, UK). The relative fluorescence intensity of HIF1α/DAPI was quantified using the ImageJ software.

### Intracellular calcium concentration [Ca^2+^]_i_ measurement

Changes in [Ca^2+^]_i_ were observed using Fluo 3-AM (Invitrogen, #F1242) dissolved in DMSO. The cells were washed once with PBS, incubated in PBS containing Fluo 3-AM (4 μM) with 5% CO_2_ at 37°C for 30 min, then washed once with PBS and scanned every second using Eclipse Ts2 fluorescence microscopy (Nikon). The fluorescence was excited at 488 nm, and the emitted light was read at 515 nm. In order to verify the assay, A23187 was applied to the cells as a positive control. All [Ca^2+^]_i_ analyses were processed at a single cell level and expressed as the relative fluorescence intensity.

### *In situ* proximity ligation assay (PLA)

HIF1α/BICD1 and HIF1α/Dynein IC interactions were detected *in situ* using Duolink II secondary antibodies and detection kits (Sigma-Aldrich, #DUO92001, #DUO92005, and #DUO92008) according to the manufacturer’s instructions. Briefly, PLA probes and primary antibodies against anti-HIF1α, anti-BICD1, and anti-Dynein IC were applied to fixed cells. Then Duolink secondary antibodies were added. These secondary antibodies were ligated together to make a closed circle by the Duolink ligation solution if the antibodies were in close proximity ( < 40 nm). Polymerase and amplification buffers were added to amplify the positive signal (red dot) of the exiting closed circle and detected by SRRF microscopy. The nucleus was counterstained using DAPI.

### Co-immunoprecipitation

UCB-MSCs were lysed with co-immunoprecipitation lysis buffer (20 mM Tris–HCl pH 8.0, 137 mM NaCl, 1% Nonidet P-40, and 2 mM EDTA) with a protease inhibitor cocktail and incubated for 30 min on ice. Protein concentrations were determined using a BCA quantification assay (Thermo Fisher Scientific, #23225). BICD1 or rabbit IgG antibodies were immobilized with protein G magnetic beads (Sure Beads, BioRad, CA, USA, #161-4021). The immobilized magnetic beads were incubated with cell lysates for 6 h at 4°C. Washed beads were eluted with 20 mM glycine buffer (pH 2.0) for 5 min and neutralized with 1 M phosphate buffer. Protein samples were then boiled at 100°C for 5 min.

### Statistical analysis

All quantitative data were presented as mean ± standard error of mean (S.E.M). Data were analyzed using the SigmaPlot 12 software. The sample sizes for animal studies were determined by previous report^44^ and SigmaPlot 12 software. Comparisons between two experimental groups were performed using the two-tailed Student’s *t*-test. The means of multiple experimental groups were compared using One-way ANOVA, followed by the Student-Newman-Keuls’s test for multiple comparisons. A level of *p* < 0.05 was considered statistically significant.

## Results

### Effect of cP1P on mitochondrial ROS accumulation and MSC survival under hypoxia

**Fig. 1:**
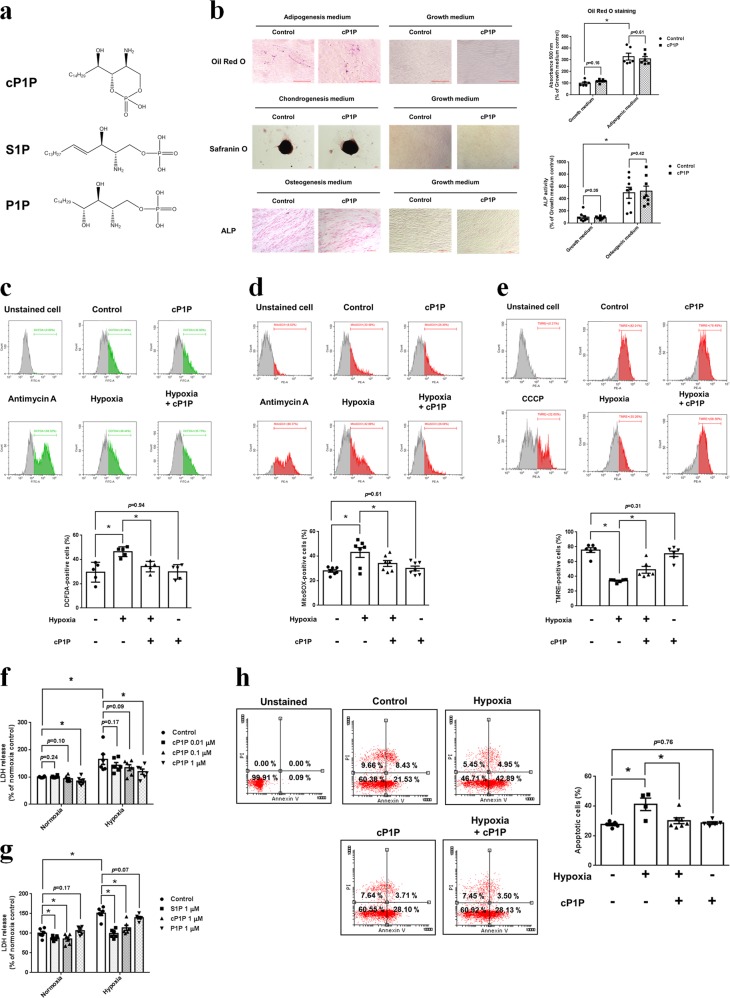
Effect of cP1P on mitochondrial ROS accumulation and apoptosis of UCB-MSCs under hypoxia. **a** Structural formulas of cP1P, S1P, and P1P are shown. **b** Differentiated adipocytes, chondrocytes, and osteocytes from UCB-MSCs were fixed and stained with Oil Red O, safranin O, or ALP, respectively. Lipid accumulation in undifferentiated and adipo-differentiated UCB-MSCs was assessed by quantification of absorbance at 500 nm in de-stained Oil Red O with isopropyl alcohol. *n* = 6. The ALP activities of undifferentiated and osteo-differentiated UCB-MSCs were measured by using commercial kit. *n* = 8. **c**–**e** UCB-MSCs were pretreated with cP1P (1 μM) for 30 min prior to hypoxia incubation for 72 h. **c** Antimycin A (10 μM) was treated to UCB-MSCs for 2 h. Intracellular ROS level was assessed by DCFDA staining. DCFDA-positive cells were measured by using flow cytometer. *n* = 5. **d** Antimycin A (10 μM) was treated to UCB-MSCs for 2 h. Mitochondrial ROS level in UCB-MSC was assessed by MitoSOX staining. MitoSOX-positive cells were measured by using flow cytometer. *n* = 7. **e** Carbonyl cyanide m-chlorophenyl hydrazone (CCCP, 10 μM) was treated to UCB-MSCs for 4 h. Mitochondrial membrane potential was assessed by TMRE staining. TMRE-positive cells were measured by using flow cytometer. *n* = 6. Quantitative data are presented as a mean ± S.E.M with scatter plots. * indicates *p* < 0.05. **f**–**h** UCB-MSCs were pretreated with cP1P (0.01, 0.1, or 1 μM), S1P (1 μM), or P1P (1 μM) prior to hypoxia incubation for 72 h. **f**, **g** LDH in UCB-MSC-conditioned medium was detected by using commercial kit. *n* = 6. **h** The percentages of AnnexinV-positive apoptotic cells were measured by flow cytometer. Annexin V-positive/PI-positive cells and Annexin V-positive/PI-negative cells were considered apoptotic cells. *n* = 4–7. All flow cytometer images are representative. Quantitative data are presented as a mean ± S.E.M with scatter plots. * indicates *p* < 0.05

To identify cP1P’s biological characteristics and MSC survival in oxidative stress, we treated cP1P or other sphingosine metabolites, such as S1P and P1P, to UCB-MSCs under hypoxia (Fig. 1a). First, cP1P did not affect the UCB-MSCs potential of differentiation into adipocytes, chondrocytes, and osteocytes (Fig. 1b). Under hypoxia, pretreating cP1P decreased the percentages of DCFDA- and MitoSOX-positive cells compared with nontreated UCB-MSCs (Fig. 1c, d). Hypoxia-related decrease in TMRE-positive cells was reversed by cP1P (Fig. 1e) However, neither cP1P nor S1P affected the antioxidant enzymes inducing the expression of the Nrf2 and the acetylation and expression of SOD2 (Supplementary fig. S1a). After 72 h of hypoxia, cP1P decreased the release of LDH in a dose-dependent manner (Fig. 1f). Hypoxia-induced LDH release was significantly decreased by both cP1P and S1P, but not P1P (Fig. 1g). Annexin V/PI analysis data showed that the percentage of apoptotic UCB-MSCs with hypoxia and cP1P was lower than that of UCB-MSCs with hypoxia alone (Fig. 1h). These results indicate that, similar to S1P, cP1P induces the anti-apoptosis of UCB-MSCs exposed to hypoxia. The proliferation rate of UCB-MSCs treated with cP1P or S1P for 48 h is higher than that of nontreated or P1P-treated UCB-MSCs (Supplementary fig. S1b). The dosages of 0.1 and 1 μM of cP1P treatment significantly increased UCB-MSC proliferation both under normoxia and hypoxia for 72 h (Supplementary fig. S1c). All cP1P, S1P, and P1P increased UCB-MSC migration (Supplementary fig. S1d). Collectively, these data suggest that cP1P stimulates UCB-MSC proliferation, migration, and anti-apoptosis, with maintenance of multiple lineage differentiation potential.

### Regulatory effect of cP1P on glucose metabolism

**Fig. 2:**
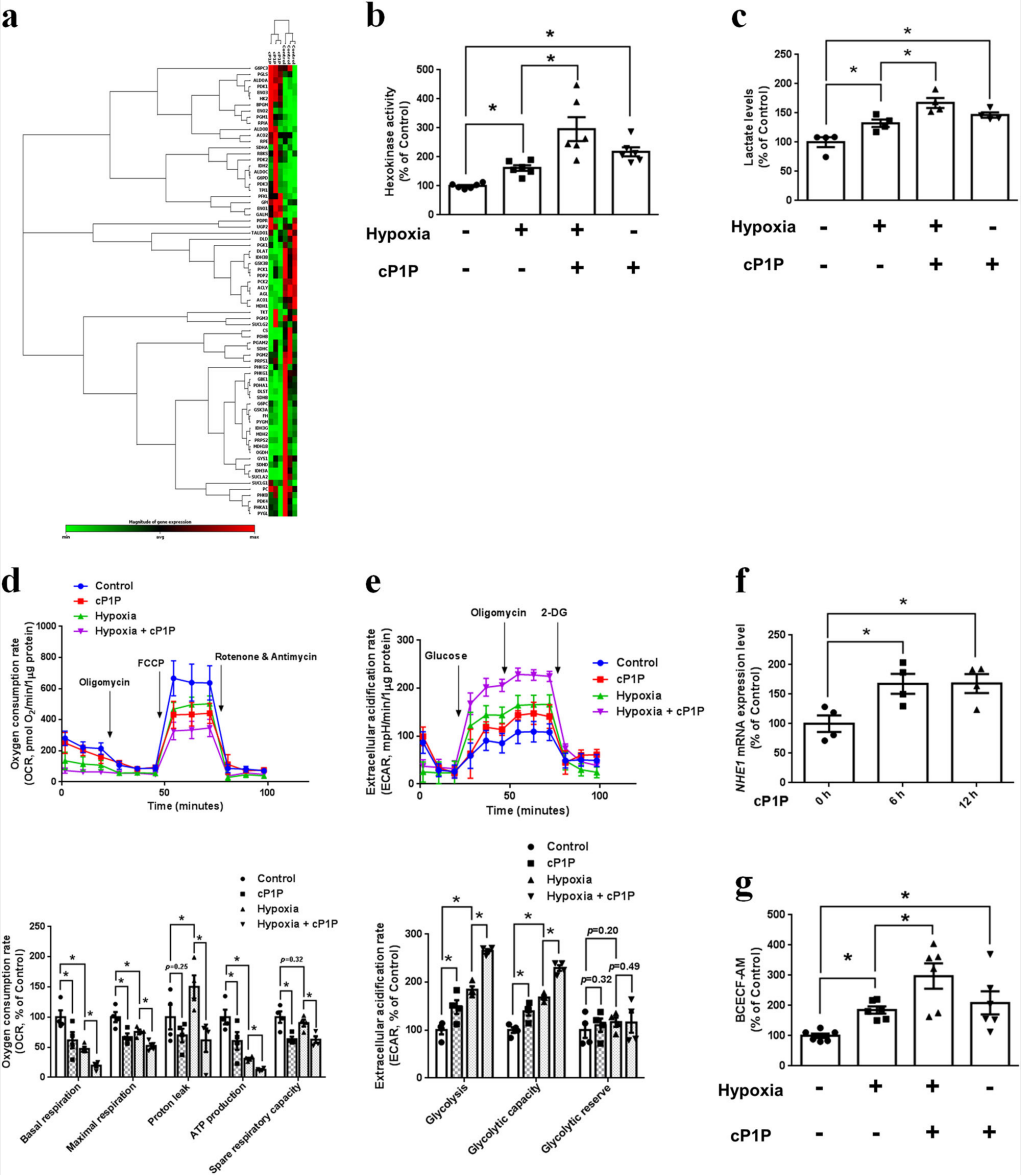
Effect of cP1P on glucose metabolism in UCB-MSCs. **a**, **b** UCB-MSCs were incubated with cP1P (1 μM) for 24 h. **a** The mRNA expression levels of glucose metabolism-associated genes were assessed by RT2 Profiler PCR array, heat maps with hierarchical clustering shown in left panel were acquired by using the GeneGlobe Data Analysis Center on Qiagen website (http://www.qiagen.com/kr/shop/genes-and-pathways/data-analysis-center-overview-page/). *n* = 3. **b** Hexokinase activity was measured by commercial kit. *n* = 6. **c** UCB-MSCs were pretreated with cP1P (1 μM) for 30 min prior to normoxia or hypoxia incubation for 48 h. Intracellular lactate levels in UCB-MSCs were measured by commercial kit. *n* = 4. **d**, **e** UCB-MSCs were incubated with cP1P (1 μM) for 24 h. **d** OCR changes under mitochondrial stress test were assessed by Seahorse XF24 Extracellular Flux Analyzer. Quantitative data of basal respiration, maximal respiration, proton leak, ATP production, and spare respiratory capacity are shown in bottom panel. *n* = 4. **e** ECAR changes under glycolysis stress test were assessed by Seahorse XF24 Extracellular Flux Analyzer. Quantitative data of glycolysis, glycolytic capacity, and glycolytic reserve are shown in bottom panel. *n* = 4. **f** *NHE1* mRNA expressions in UCB-MSCs treated with cP1P (1 μM) for 0, 6, or 12 h. *n* = 4. Gene expression levels were normalized by *18* *S rRNA* expression levels. **g** UCB-MSCs were incubated with cP1P (1 μM) for 24 h. Intracellular alkalization was measured by using BCECF-AM staining. *n* = 6. Quantitative data are presented as a mean ± S.E.M with scatter plots. * indicates *p* < 0.05

To determine the effect of cP1P on glucose metabolism, we investigated the glucose metabolism–associated gene mRNA expression, hexokinase activity, and lactate levels in UCB-MSCs with or without cP1P under normoxia and hypoxia. PCR array data revealed the upregulation of glycolysis-associated expression of genes (*BPGM*, *GALM*, *HK2*, *ENO3*, *ALDOA*, *ENO1*, and *PGM1*) and the downregulation of tricarboxylic acid (TCA) cycle-associated expression of genes (*PDHA1*, *MDH1*, *ALCY*, *CS*, and *ACO1*) by cP1P (Fig. 2a, Supplementary Tables S4 and S5). Consistently, cP1P increased hexokinase activity and intracellular lactate levels in UCB-MSCs under both normoxia and hypoxia (Fig. 2b, c). OCR data showed that cP1P decreased basal respiration, maximal respiration, ATP production, and spare respiratory capacity in UCB-MSCs under both normoxia and hypoxia. Hypoxia decreased basal respiration, maximal respiration, and ATP production, but it increased proton leak in UCB-MSCs (Fig. 2d). ECAR data showed that both cP1P treatment and hypoxia incubation stimulated glycolysis and glycolytic capacity but not glycolytic reserve (Fig. 2e). cP1P stimulates *NHE1* mRNA expression in a time-dependent manner, and increased intracellular alkalization in UCB-MSCs under normoxia and hypoxia (Fig. 2f, g). Taken together, we suggested that cP1P decreases the oxygen availability and oxidative phosphorylation and increases the glycolytic flux via switching the glucose metabolism by glycolysis in UCB-MSCs under both normoxia and hypoxia.

### Role of cP1P-induced HIF1α in therapeutic potential of MSC

**Fig. 3:**
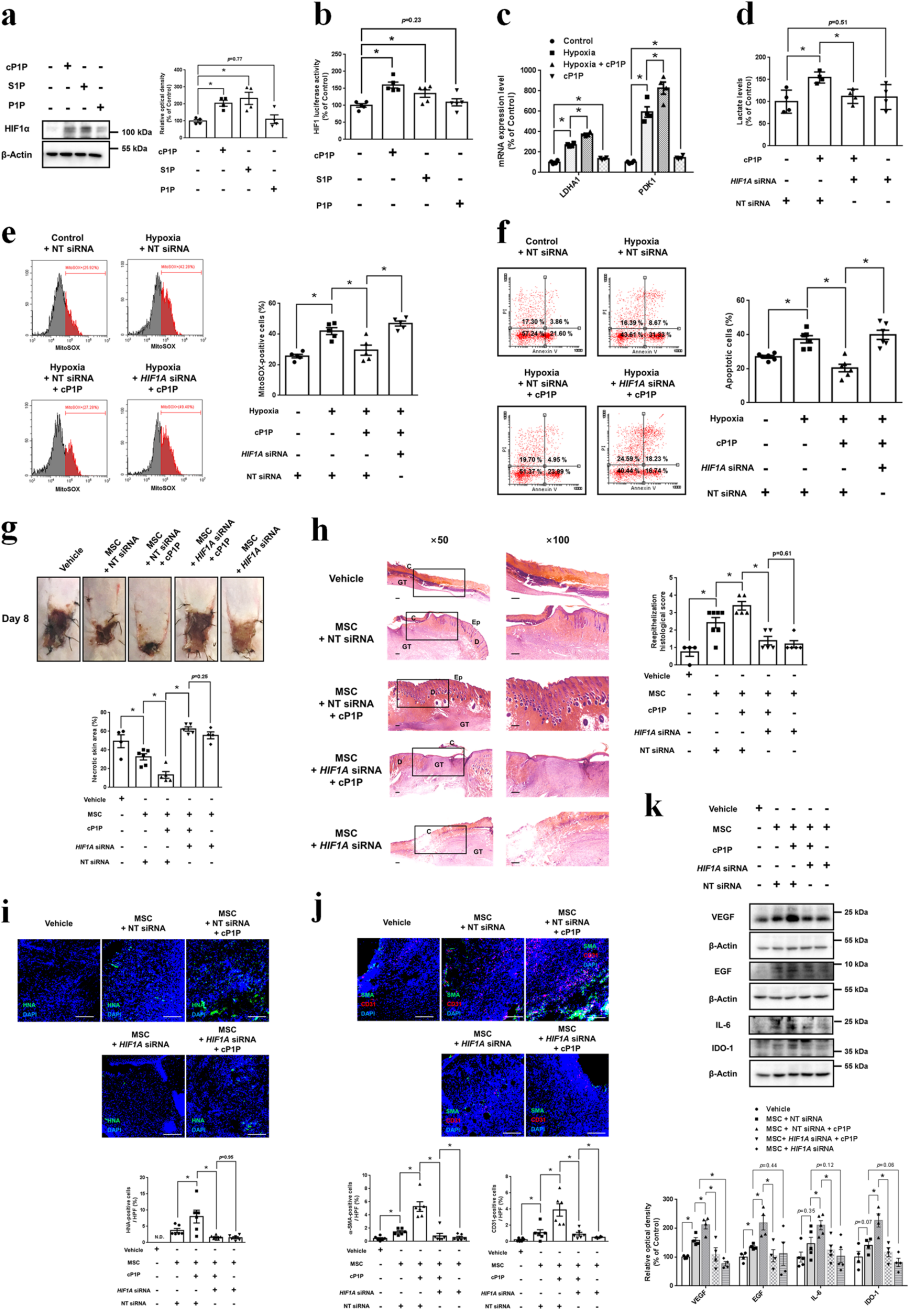
Role of cP1P-induced HIF1α in apoptosis and therapeutic potential of UCB-MSCs. **a**, **b** UCB-MSCs were treated with cP1P (1 μM), S1P (1 μM), or P1P (1 μM) for 24 h. **a** The protein expressions of HIF1α and β-Actin were detected by western blotting. *n* = 4. **b** HIF1 activities were assessed by dual luciferase reporter assay. *n* = 5. **c** UCB-MSCs were pretreated cP1P (1 μM) prior to hypoxia incubation for 48 h. The mRNA expressions of *LDHA* and *PDK1* in UCB-MSCs were normalized by *18* *S rRNA* expression levels. *n* = 4. **d** NT or *HIF1A* siRNA was transfected to UCB-MSCs for 24 h prior to cP1P (1 μM) treatment for 48 h. Intracellular lactate levels in UCB-MSCs were measured by using commercial kit. *n* = 4. **e**, **f** NT or *HIF1A* siRNA-transfected UCB-MSCs pretreated with cP1P (1 μM) for 30 min prior to hypoxia treatment for 72 h. **e** MitoSox-positive cells were measured by using flow cytometer. *n* = 6. **f** The percentages of AnnexinV-positive apoptotic cells were measured by flow cytometer. Annexin V-positive/PI-positive cells and Annexin V-positive/PI-negative cells were considered apoptotic cells. *n* = 6. **g**–**k** Mouse skin flap surgery with vehicle, NT-siRNA-transfected UCB-MSCs or *HIF1A* siRNA-transfected UCB-MSCs with or without cP1P pretreatment for 24 h prior to transplantation was performed as described in Materials & Methods section. **g** Necrotic regions of skin flaps at post-injection day 8 were compared with those at post-injection day 0. Representative gross images are shown in upper panel. *n* = 4–6. **h** Representative histological images are shown in left panel. Tissue samples at post-injection day 11 were fixed and stained with hematoxylin and eosin. Scale bars are 200 μm (Magnification, × 50 or × 100). C crust, GT granulated tissue, D dermis, Ep epidermis. **i**, **j** Tissue slide samples were immunostained with HNA, CD31, or α-SMA-specific antibodies. The percentages of HNA, CD31, or α-SMA-positive cells in high power field (HPF) were analyzed by using Metamorph software. Scale bars are 100 μm. Magnification × 100. *n* = 6. All gross and immunofluorescence images are representative. **k** The protein expressions of VEGF, EGF, IL-6, IDO-1, and β-Actin in tissue samples were measured by western blotting. *n* = 4–5. All blot and immunofluorescence images are representative. Quantitative data are presented as a mean ± S.E.M with scatter plots. * indicates *p* < 0.05. N.D. indicates not detected

To identify the metabolic regulator of cP1P-induced glycolysis, we analyzed the HIF1α expression and HIF1 activity in UCB-MSCs with cP1P, S1P, and P1P. While both cP1P and S1P induce HIF1α expression and HIF1 activity, P1P did not affect HIF1 luciferase activity (Fig. 3a, b). The total and nuclear expression levels of HIF1α in cP1P-pretreated UCB-MSCs were higher than vehicle-pretreated UCB-MSCs with or without hypoxia (Supplementary fig. S2a, b). Next, we found that cP1P increased the mRNA expressions of HIF1-targeted glycolysis genes, such as *LDHA* and *PDK1*, in UCB-MSCs under normoxia and hypoxia (Fig. 3c). *HIF1A* silencing reversed the cP1P-increased intracellular lactate and –decreased mitochondrial ROS levels (Fig. 3d, e), and it abolished the cP1P-increased survival rate of UCB-MSCs under hypoxia (Fig. 3f and Supplementary fig. S3). These findings suggest that cP1P-stimulated HIF1α is a key factor regulating the glycolysis metabolism and reversing the apoptosis of UCB-MSCs under hypoxia.

Next, we investigated the effect of cP1P and *HIF1A* silencing on the therapeutic potential of transplanted UCB-MSCs into the mouse skin flap model. Eight days after UCB-MSCs transplantation into the ischemic skin flap, the necrotic area of the *HIF1A* siRNA-transfected UCB-MSCs with cP1P transplantation group was significantly greater than that of NT siRNA-transfected UCB-MSCs with cP1P transplantation group (Fig. 3g). NT siRNA-transfected UCB-MSCs with cP1P had the highest histological score among all groups (Fig. 3h). The percentage of HNA, the marker of transplanted UCB-MSCs-positive cells in NT siRNA-transfected UCB-MSCs with cP1P group was significantly higher than NT siRNA-transfected UCB-MSCs group with vehicle pretreatment (Fig. 3i). Consistently, the percentages of the pan-endothelial marker CD31- and the myofibroblast marker α-SMA-positive cells at skin-flap tissue had a similar pattern in the experimental group, given the NT siRNA-transfected UCB-MSCs with or without cP1P and *HIF1A* siRNA-transfected UCB-MSCs with cP1P (Fig. 3j). In addition, the expression of cytokines involved in wound healing process and immune-modulation, such as VEGF, EGF, IL-6, and IDO-1, in NT siRNA-transfected UCB-MSCs with cP1P transplantation group was the highest among all experimental groups (Fig. 3k). However, cP1P did not affect the expression levels of VEGF, EGF, IL-6, and IDO-1 in UCB-MSCs (Supplementary fig. S4). These findings indicate that the survival rate of transplanted UCB-MSCs is an increasing factor in the levels of those cytokines in the skin during wound healing process.

### Involvement of S1PR1-dependent PKCα/mTOR pathway in cP1P-stimulated HIF1α translation

**Fig. 4:**
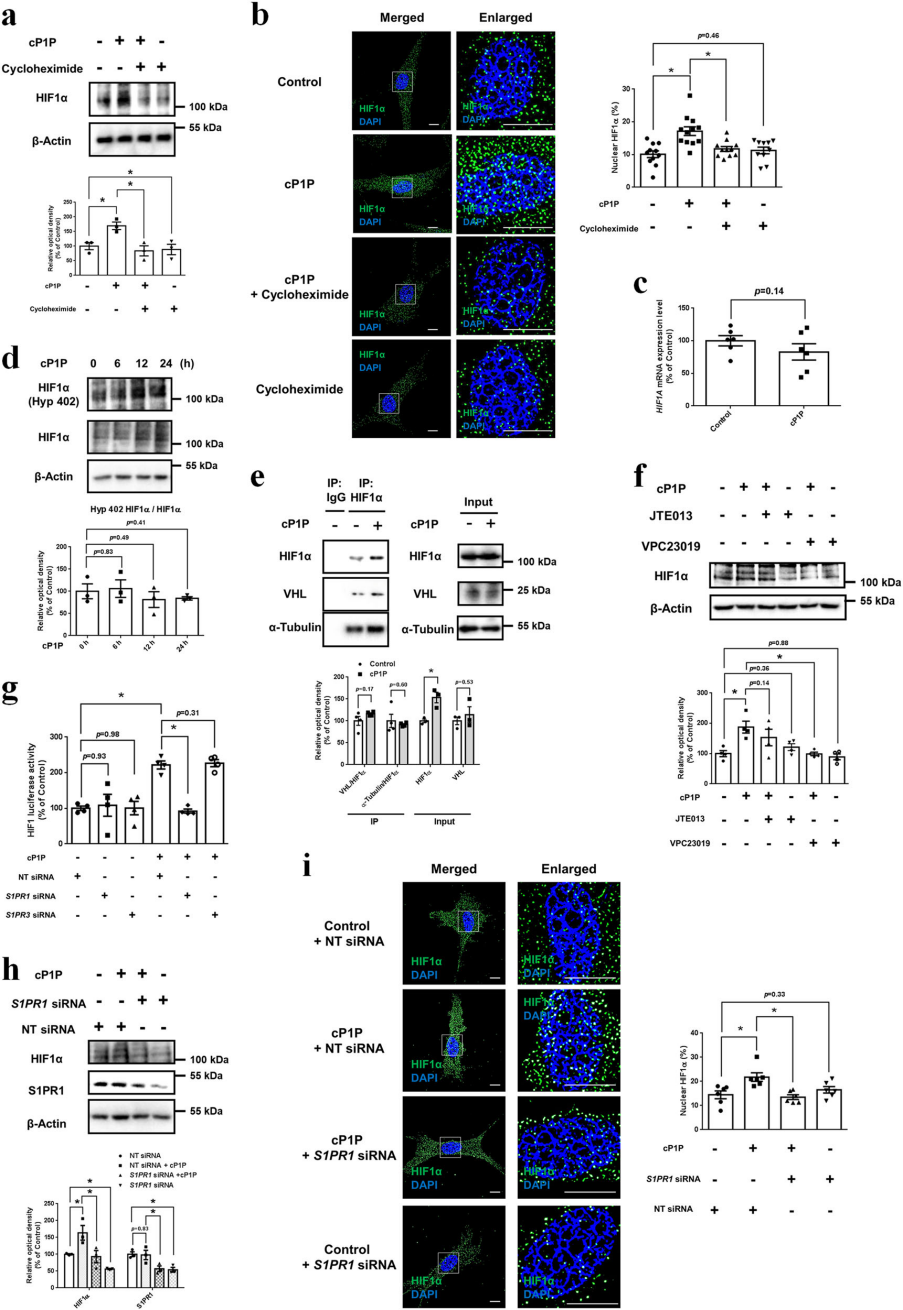
Role of S1PR1 in cP1P-induced HIF1α translation. **a**, **b** UCB-MSCs were pretreated with cycloheximide (10 μM) for 30 min prior to cP1P (1 μM) treatment for 24 h. **a** The protein expressions of HIF1α and β-Actin were detected by western blotting. *n* = 3. **b** Cells were immunostained with HIF1α -specific antibody. *n* = 10–12. Scale bars are 8 μm. Magnification × 1,000 (left panel). **c**, **e** UCB-MSCs were treated with cP1P (1 μM) for 24 h. **c** *HIF1A* mRNA expression level was normalized by *18* *S rRNA* expression level. *n* = 6. **d** UCB-MSCs were incubated with cP1P (1 μM) for 0, 6, 12, or 24 h. The protein expression of prolyl hydroxylated HIF1α, HIF1α, and β-Actin were detected by western blotting. *n* = 3. **e** Co-immunoprecipitation of VHL and α-Tubulin with IgG and HIF1α antibodies are shown in left panel. Total protein expressions of HIF1α, VHL, and α-Tubulin in lysates are shown in right panel. *n* = 4. **f** UCB-MSCs were pretreated with JTE013 (10 μM) or VPC23019 (1 μM) for 30 min prior to cP1P (1 μM) treatment for 24 h. The protein expressions of HIF1α and β-Actin were detected by western blotting. *n* = 4. **g**–**i** UCB-MSCs were transfected with *S1PR1*, *S1PR3*, or NT siRNA for 24 h prior to cP1P (1 μM) treatment for 24 h. **g** HIF1 activities were assessed by dual luciferase reporter assay. *n* = 4. **h** The protein expressions of HIF1α, S1PR1, and β-Actin were detected by western blotting. *n* = 3. **i** UCB-MSCs were immunostained with HIF1α-specific antibody. *n* = 6. Scale bars are 8 μm. Magnification × 1,000 (left panel). All blot and immunofluorescence images are representative. Quantitative data are presented as a mean ± S.E.M with scatter plots. * indicates *p* < 0.05

Next, we investigated its effect on HIF1α transcription, translation, and prolyl hydroxylation. cP1P-induced HIF1α expression was inhibited by pretreatment with the translation inhibitor cycloheximide (Fig. 4a). Cycloheximide decreased the percentage of nuclear HIF1α (Fig. 4b). However, no statistically significant difference was found between control and cP1P on the mRNA expression and prolyl hydroxylation (Hyp402) of HIF1α (Fig. 4c, d). Interaction between VHL and HIF1α was not changed by cP1P (Fig. 4e). The HIF1α protein level in cycloheximide-treated UCB-MSCs with or without cP1P was decreased in a time-dependent manner (Supplementary fig. S5). These findings indicate that cP1P induced HIF1α through translation but not transcription and prolyl hydroxylation-dependent stabilization. Furthermore, cP1P-induced HIF1α was abolished by pretreatment of S1PR1/S1PR3 inhibitor VPC23019 but not S1PR2 inhibitor JTE013 (Fig. 4f). cP1P did not increase HIF1 luciferase activity in *S1PR1*-silenced UCB-MSCs (Fig. 4g). *S1PR1* silencing suppressed both total HIF1α expression and the percentage of nuclear HIF1α (Fig. 4h, i). Hypoxia decreased the expression level of *S1PR4* mRNA without affecting that of other S1PRs’ mRNA (Supplementary fig. S6). These results suggest that cP1P increases HIF1α expression in a translation-dependent manner via the S1PR1 pathway.

**Fig. 5:**
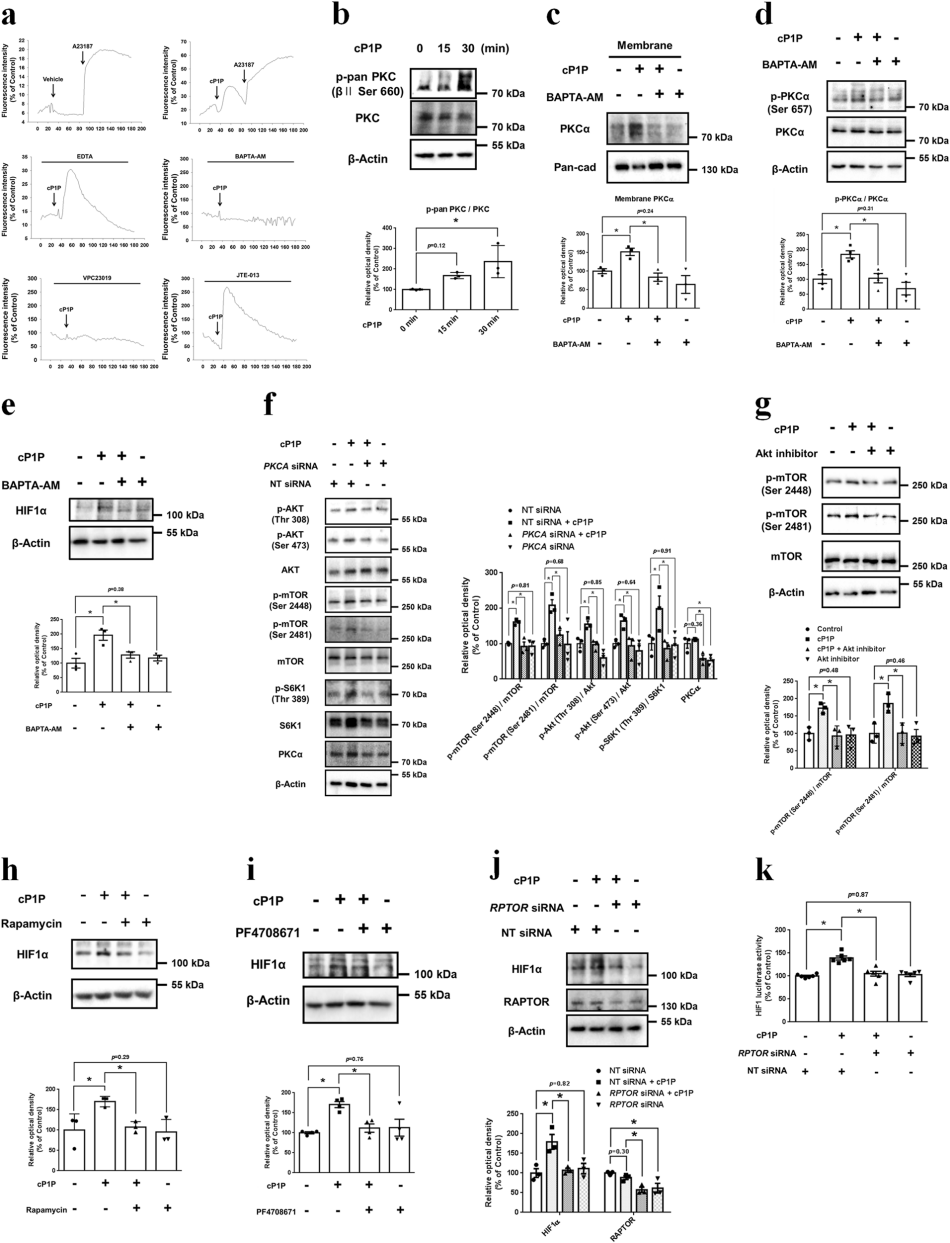
Involvement of calcium-dependent PKCα/mTOR pathway in cP1P-induced HIF1α expression. **a** UCB-MSCs were pretreated with EDTA (2 mM), BAPTA-AM (10 μM), VPC23019 (1 μM), or JTE013 (10 μM), and incubated with fluo-3-AM for 30 min prior to calcium influx measurement with pretreatment of vehicle, cP1P (1 μM), or A23187 (1 μM). **b** UCB-MSCs were treated with cP1P (1 μM) for 0, 15, or 30 min. p-pan PKC (βII Ser660), PKC, and β-Actin were detected by western blotting. *n* = 3. **c**, **d** UCB-MSCs were pretreated with BAPTA-AM (10 μM) for 30 min prior to cP1P (1 μM) for 30 min. **c** PKCα and pan-cad protein expressions in membrane fraction samples were detected by western blotting. *n* = 3. **d** Total protein expressions of p-PKCα, PKCα, and β-Actin were detected by western blotting. *n* = 4. **e** UCB-MSCs were pretreated with BAPTA-AM (10 μM) for 30 min prior to cP1P (1 μM) for 24 h. Total protein expression levels of HIF1α and β-Actin were detected by western blotting. *n* = 3. **f** UCB-MSCs were transfected with *PKCA* or NT siRNA for 24 h prior to cP1P (1 μM) for 30 min. The protein expressions of p-Akt (Thr308), p-Akt (Ser473), Akt, p-mTOR (Ser2448), p-mTOR (Ser2481), mTOR, p-S6K1 (Thr389), S6K1, PKCα, and β-Actin were detected by western blotting. *n* = 3. **g** UCB-MSCs were pretreated with Akt inhibitor (2 μM) for 30 min prior to cP1P (1 μM) for 30 min. p-mTOR (Ser2448), p-mTOR (Ser2481), mTOR, and β-Actin were detected by western blotting. *n* = 3. **h**, **i** UCB-MSCs were pretreated with rapamycin (100 nM) or PF4708671 (10 μM) for 30 min prior to cP1P (1 μM) for 24 h. HIF1α and β-Actin protein expressions were detected by western blotting. *n* = 3–4. **j**, **k** UCB-MSCs were transfected with *RPTOR* or NT siRNA for 24 h prior to cP1P (1 μM) for 24 h. **j** RAPTOR, HIF1α and β-Actin protein expressions were detected by western blotting. *n* = 3. **k** HIF1 activities were assessed by dual luciferase reporter assay. *n* = 6. All blot images are representative. Quantitative data are presented as a mean ± S.E.M with scatter plots. * indicates *p* < 0.05

We next investigated the effect of cP1P on calcium signaling and PKC activation. Our results showed that cP1P increased the intracellular calcium level, although it did not affect the calcium levels of UCB-MSCs pretreated with BAPTA-AM and VPC23019 (Fig. 5a). These data indicate that cP1P stimulates intracellular calcium release via S1PR1/3 signaling. In addition, cP1P increased the phosphorylation of PKC (Ser660), Akt (Thr308 and Ser473), and GSK3β (Ser9) (Fig. 5b and Supplementary fig. S7a). cP1P stimulated the membrane localization of PKCα, but not PKCδ and PKCε, which were reduced by BAPTA-AM (Supplementary fig. S7b and Fig. 5c). BAPTA-AM pretreatment inhibited cP1P-induced PKCα phosphorylation (Ser657) and HIF1α expression (Fig. 5d, e). Furthermore, we studied the role of calcium-activated PKCα in an Akt/mTOR/S6K1 pathway in cP1P-treated UCB-MSCs. BAPTA-AM pretreatment suppressed the cP1P-induced phosphorylations of Akt, mTOR and S6K1, whereas cP1P did not affect the phosphorylation of Akt, mTOR or S6K1 in *PKCA* siRNA-transfected UCB-MSCs (Supplementary fig. S8 and Fig. 5f). Akt inhibitor suppressed the cP1P-induced phosphorylation of mTOR at Ser2448 and Ser2481 residues (Fig. 5g). Pretreatment of mTOR inhibitor rapamycin, S6K1 inhibitor PF4708671, or *RPTOR* siRNA abolished cP1P-induced HIF1α expression (Fig. 5h–j). Results from the dual luciferase reporter assay showed that cP1P-activated HIF1 luciferase activity was suppressed by *RPTOR* silencing (Fig. 5k). In addition, cP1P did not increase HIF1 expression in *RPTOR* knockout SK-N-MCs neuroblastoma cells (Supplementary fig. S9). Taken together, these results indicate that the calcium-activated PKCα/mTOR pathway is essential for cP1P-induced HIF1α translation in UCB-MSCs.

### Role of cP1P-induced BICD1 in HIF1α nuclear translocation

**Fig. 6:**
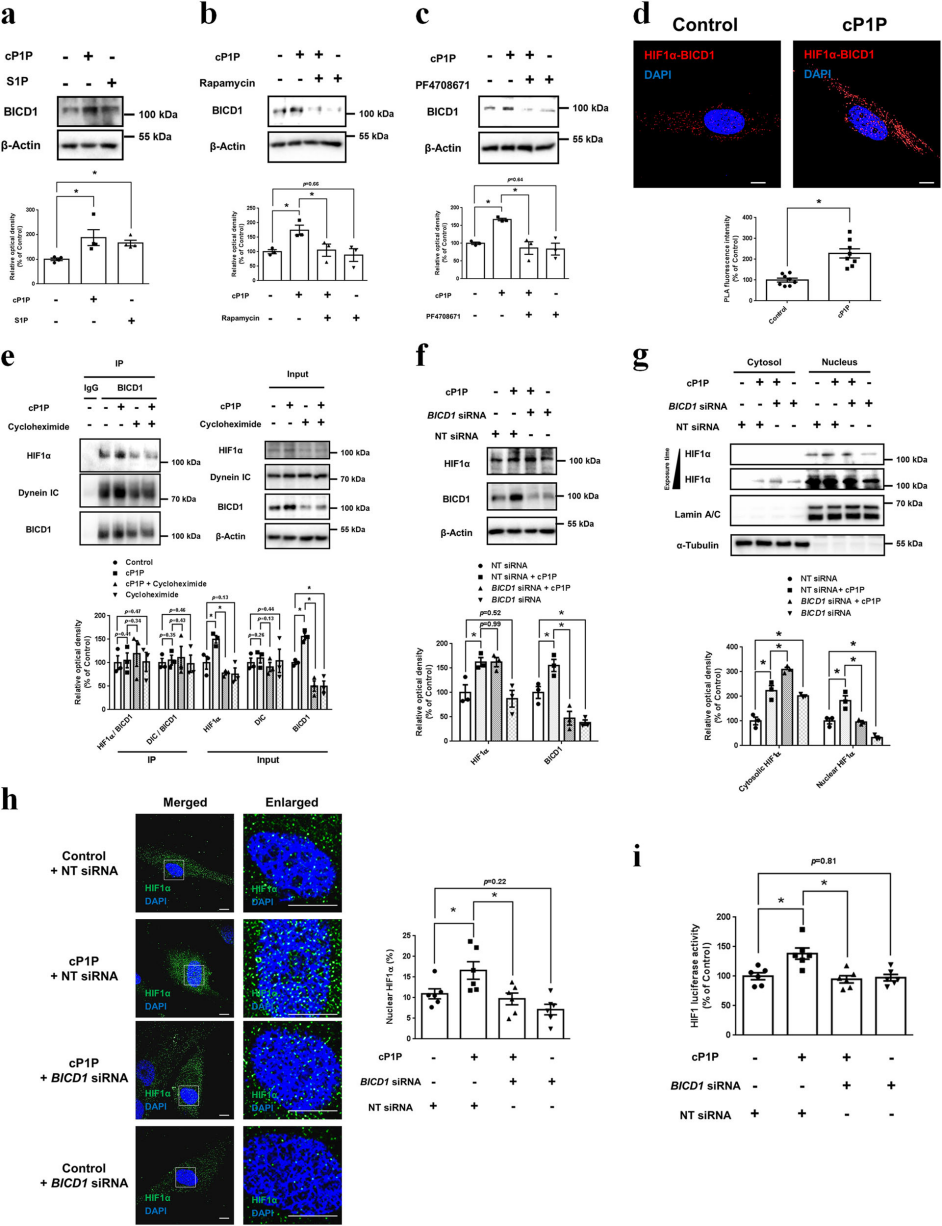
Role of BICD1 in cP1P-induced HIF1α expression. **a** UCB-MSCs were incubated with cP1P (1 μM) or S1P (1 μM) for 24 h. The protein expressions of BICD1 and β-Actin were detected by western blotting. *n* = 4. **b**, **c** UCB-MSCs were pretreated with rapamycin (100 nM) or PF4708671 (10 μM) for 30 min prior to cP1P (1 μM) for 24 h. BICD1 and β-Actin protein expressions were detected by western blotting. *n* = 3. **d** Interaction between HIF1α and BICD1 (HIF1α-BICD1, red) in UCB-MSCs with or without cP1P (1 μM) treatment for 24 h was assessed by PLA assay. Scale bars are 8 μm. Magnification × 1,000. *n* = 8. **e** UCB-MSCs were pretreated with cycloheximide (10 μM) for 30 min prior to cP1P (1 μM) for 24 h. Co-immunoprecipitation of HIF1α and Dynein IC with IgG and BICD1 antibodies are shown in left panel. Total protein expressions of HIF1α, Dynein IC, BICD1, and β-Actin in lysates are shown in right panel. *n* = 3. **f**–**i** UCB-MSCs were transfected with *BICD1* or NT siRNA for 24 h prior to cP1P (1 μM) treatment for 24 h. **f** Total protein expressions of HIF1α, BICD1, and β-Actin were detected by western blotting. *n* = 3. **g** Nuclear and cytosolic protein expressions of HIF1α, lamin A/C, and α-Tubulin were detected by western blotting. *n* = 3. **h** UCB-MSCs were immunostained with HIF1α-specific antibody. *n* = 6. Scale bars are 8 μm. Magnification × 1,000 (left panel). **i** HIF1 activities were assessed by dual luciferase reporter assay. *n* = 6. All blot and immunofluorescence images are representative. Quantitative data are presented as a mean ± S.E.M with scatter plots. * indicates *p* < 0.05

We also found that both cP1P and S1P significantly stimulate BICD1 expression (Fig. 6a). BICD1 induction by cP1P was abolished by rapamycin or PF4708671 pretreatment, indicating that cP1P-activated mTOR/S6K1 may potentially lead to BICD1-mediated HIF1α nuclear translocation (Fig. 6b, c). cP1P did not induce BICD1 expression in *RPTOR*-deficient SK-N-MCs (Supplementary fig. S9). Furthermore, we investigated the effect of cP1P on the interaction between HIF1α and BICD1. HIF1α-BICD1 PLA fluorescence intensity in cP1P-treated UCB-MSCs increased to 228% (Fig. 6d). However, cP1P did not change the ratio of HIF1α and dynein IC to BICD1 but increased the total expressions of HIF1α, dynein IC, and BICD1 (Fig. 6e). HIF1α/BICD1 complex formation was significantly decreased by cycloheximide pretreatment (Fig. 6e). *BICD1* silencing did not influence total HIF1α expression but decreased cP1P-induced nuclear expression of HIF1α, the percentage of nuclear HIF1α and the HIF1 luciferase activity (Fig. 6f–i). Collectively, these results indicate that BICD1 expression induced by cP1P plays a key role in HIF1α nuclear translocation for HIF1 activation.

## Discussion

We highlight the regulatory effect of cP1P on HIF1α expression and nuclear translocation for the enhancement of therapeutic potential of MSCs under hypoxia. Previous studies have reported that mitochondrial ROS is a major risk factor of apoptosis and immunopotency suppression in MSCs exposed to hypoxia^43,45–47^. Aberrant control of mitochondrial ROS level impairs the integrity of mitochondrial membranes, thereby activating the caspase-dependent apoptosis signaling pathway^48^. We first showed that cP1P treatment reduced mitochondrial ROS, preventing the loss of mitochondrial membrane potential and inhibiting apoptosis in hypoxia-exposed MSC. Previous studies have demonstrated that lymphatic endothelial S1P stimulates mitochondrial function and protects from oxidative phosphorylation impairment, subsequently leading to anti-apoptosis of naïve T cells^49^. Since a survival effect was observed in cP1P- or S1P-treated MSCs under hypoxia, we suggest that, similar to S1P, cP1P exerts an anti-oxidative potential in MSCs through the regulation of mitochondrial ROS accumulation.

Recent studies have shown the regulatory role of S1P in HIF1α expression and glycolysis. Sphingosine kinase-stimulated S1P production activated by hypoxia increased HIF1α expression^50^. S1P production by hypoxia stimulated the glycolytic flux in erythrocytes through the release of a membrane-bound glycolytic enzyme, which is critical for the upregulating of hemoglobin’s oxygen release capacity in erythrocytes^26,27^. In addition to the HIF1-regulated S1P metabolism, inhibition of sphingosine kinase 2, the isotype of mitochondrial S1P-producing enzyme, reduced oxidative stress through the suppression of Nrf2 accumulation, SOD2 expression, and mitochondrial respiration^51,52^. However, our data revealed that cP1P did not affect Nrf2 accumulation or SOD2 expression and acetylation. Therefore, we focused on the regulatory effect of cP1P on HIF1α-regulated glucose metabolism. We also found that cP1P stimulates glycolytic flux by upregulating the expression of glycolysis-regulating enzymes in MSCs exposed to either normoxia or hypoxia, which depends on HIF1α. A previous study on gene regulation demonstrated that HIF1α is a key factor maintaining the survival of MSCs under hypoxia^37^. In addition, HIF1 induction by hypoxia preconditioning improves tissue regeneration and blood perfusion in ischemic tissue^25^. Consistently, we demonstrated that cP1P-induced HIF1α is important for tissue regeneration, angiogenesis, and transplanted cell survival in ischemic regions. Therefore, we propose that cP1P-induced HIF1α plays a stimulatory role for the therapeutic potential of MSC transplantation into the ischemic model.

In the present study, we demonstrated that cP1P increases the HIF1α expression level in a S1PR1-induced, translation-dependent manner. According to previous reports, S1P mainly induces HIF1α protein expression through mTOR-dependent translation but does not increase the mRNA expression level of *HIF1A* and pVHL-dependent HIF1α stabilization^38,39,50^. However, the role of S1PRs in S1P-induced HIF1α translation remains controversial. Several studies reported that S1P–increased HIF1α expression was abolished by S1PR1/S1PR3 inactivation or *S1PR3* silencing in liver hepatocellular carcinoma cells and thyroid follicular carcinoma cells, respectively^38,50^. However, another study presented S1PR2 as a normoxic HIF1α regulator in vascular endothelial and smooth muscle cells^39^. These reports suggest that the physiological role of S1PRs in S1P and cP1P-induced HIF1α translation may potentially be cell type specific. Although the regulatory effect of another S1PR activator P1P on HIF1 has been not reported, data from our dual luciferase assay revealed that P1P treatment did not significantly induce HIF1 activity. However, we found that P1P-treated MSCs exhibited increased cell migration ability under normoxia, which is consistent with a previous report^6^. Contrary to our results, a previous study demonstrated that, similar to S1P, P1P exerts an anti-oxidative potential in human dermal fibroblasts pretreated with hydrogen peroxide via the c-Jun N-terminal kinase (JNK)/Akt pathway^53^. Given that P1P’s binding affinity to S1PR4 is much higher than that of S1P^4^ and that hypoxia decreased the mRNA expression level of S1PR4 in UCB-MSCs (Supplementary fig. S3), we suggest that hypoxia decreases the susceptibility of UCB-MSCs to P1P, thereby reducing its protective effect. Therefore, previous and present findings indicate that changes in the chemical structure of P1P to cP1P increase S1PR1-activating ability, leading to a HIF1α-mediated anti-apoptotic effect on UCB-MSCs under hypoxia. However, structural and functional investigations into the binding affinity of cP1P to S1PRs will be required to deeply understand the cP1P action mechanism.

Although the detailed mechanism underlying the induction of HIF1α protein expression by S1PR1 has been poorly understood, the present mechanistic study demonstrated that cP1P-activated S1PR stimulates intracellular calcium release, thereby upregulating HIF1α translation via PKCα/mTOR signaling. It has been reported that S1PR1 has great potential for intracellular calcium mobilization in several cell types. Intracellular calcium release was suppressed in the endoplasmic reticulum of rat sensory neurons through S1PR1/3 inhibition by VPC23019 and increased in endothelial cells through S1PR1 activation by SEW2871^54,55^. In addition to calcium regulation by S1PR1, previous studies demonstrated that PKCα interacts directly with S1PR1, and the S1PR1-induced caspase-3 cleavage inhibition was abolished by PKC inhibition^56,57^. *S1PR1* siRNA transfection suppressed the ERK1/2 phosphorylation induced by S1PR1 agonists. Collectively, these findings suggest that calcium-dependent PKCα is the downstream messenger of S1PR1 activated by cP1P^58^.

Furthermore, several previous studies reported that the mTOR complex 1 (mTORC1) signaling triggered by calcium-dependent PKCα activation is Akt dependent or independent. In mammary epithelial cells, PKCα-induced signaling activates a rapamycin-insensitive companion of a mammalian target of rapamycin (RICTOR)-dependent mTORC2, which fully phosphorylates the Akt linked to mTORC1 signaling^59,60^. Meanwhile, another study showed that EGF receptor signaling activates mTORC1/ribosomal S6 (S6) pathway, suppressed by Akt inhibition^61^. However, our data showed that cP1P-activated Akt acts as an upstream regulator of the mTOR/S6K1 signaling pathway. Several researchers have reported the relationship between mTORC1 activation and HIF1α expression^32,62,63^. Activated mTORC1 drives HIF1α translation in a multifaceted manner through S6K1/S6 and eukaryotic initiation factor 4E (eIF4E) binding protein 1 (4EBP1)/eIF4E pathways^32^. In addition, the mTORC1 regulatory protein RAPTOR interacts with mTOR signaling motif in the N-terminal region of HIF1α, which is required for VHL-independent HIF1 activation through the binding to CREB binding protein (CBP)/p300, the transcriptional coactivator of HIF1^64^.

**Fig. 7:**
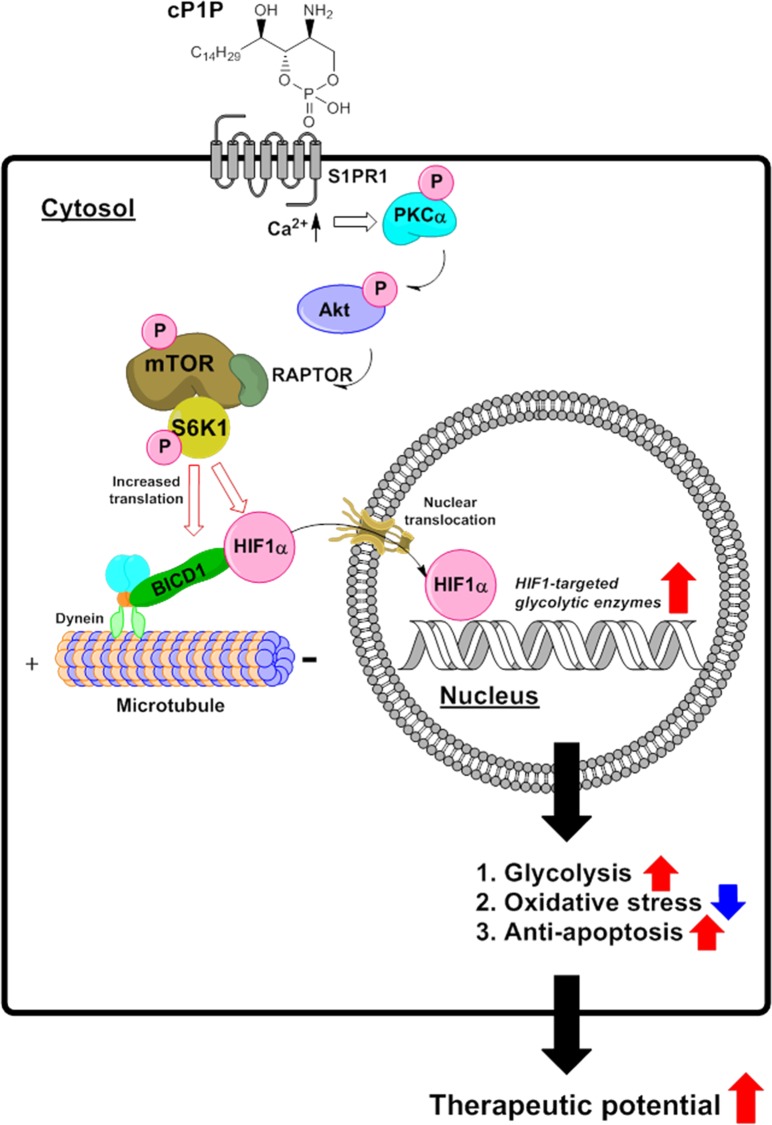
The schematic model for action mechanism of cP1P-induced therapeutic potential of UCB-MSCs. cP1P stimulates intracellular calcium release through S1PR1 activation, which leads to PKCα/Akt/mTORC1 pathway activation. Activated mTORC1 increases HIF1α and BICD1 expression in a translation-dependent manner. BICD1 mediates upregulated HIF1α nuclear translocation, which is critical for HIF1 activation. HIF1 transcriptionally stimulates glycolysis-associated enzymes mRNA expression leading to glycolytic switch, which is critical for resistance to oxidative stress and apoptosis under hypoxia. Conclusively, cP1P-stimulated glycolysis enhances therapeutic potential of UCB-MSCs through HIF1α activation

Additionally, we further demonstrated that mTOR-dependent BICD1 translation is another key factor for HIF1α nuclear translocation and HIF1 activation. A previous study reported that HIF1α nuclear trafficking is mediated by cytoplasmic dynein, the microtubule-associated motor protein^65^. The cargo-transport ability of cytoplasmic dynein depends on the interaction with dynein motor adaptor proteins, including BICD, lissencephaly 1, nuclear distribution protein E (NUDE) and NUDE-like^66,67^. BICD is a dynein adaptor protein leading to the minus end of microtubule-directed cargo transport^68^. According to previous reports, BICD has the capacity to transport various kinds of cargo, such as Rab6-dependent vesicles, viral genome, nucleus, and several proteins^69–73^. Recently, it was reported that BICD1 is an interacting partner of HIF1α-regulating dynein-mediated nuclear translocation and that hypoxia activates BICD1-induced HIF1α via the Akt/GSK3β pathway for hypoxia-induced glycolytic reprogramming of UCB-MSCs^74^. Considering that cP1P increases BICD1 expression levels, cP1P treatment can be a promising strategy for upregulation of BICD1-mediated HIF1α nuclear translocation. Indeed, BICD overexpression increased the dynein-dependent transport ability of cargo, including HIF1α^72,74^. Previous studies showed that the Akt/GSK3β pathway regulates the ability of BICD1 to interact with cargo proteins, such as HIF1α and ninein^74,75^. The present study revealed that cP1P phosphorylates both Akt and GSK3β but did not affect the percentage of nuclear HIF1α in cycloheximide-pretreated UCB-MSCs. These findings indicate that cP1P’s stimulatory effect on BICD1 translation is essential for HIF1α nuclear translocation. In conclusion, we propose that cP1P stimulates mTOR phosphorylation via the S1PR1/PKCα pathway, leading to HIF1α upregulation through S6K1-mediated translation and BICD1-mediated nuclear translocation. In addition, cP1P-activated HIF1α plays a key role in transplantation survival and therapeutic potential of UCB-MSCs via glycolytic reprogramming (Fig. 7). This study presents the first identification of cP1P as a novel metabolic regulator improving resistance against oxidative stress and transplanted MSC survival. The present study therefore provides new insights into the HIF1α regulation strategy for MSC-based therapy in regenerative medicine.

## Supplementary information

Supplementary material.
