# Supplementary material for: O-cyclic phytosphingosine-1-phosphate stimulates HIF1α-dependent glycolytic reprogramming to enhance the therapeutic potential of mesenchymal stem cells
Source: Cell Death Dis. 2019 Aug 5;10(8):590. doi: 10.1038/s41419-019-1823-7 (PMC6683124; doi:10.1038/s41419-019-1823-7)

**O-cyclic phytosphingosine-1-phosphate stimulates HIF1α-dependent glycolytic reprogramming to enhance the therapeutic potential of mesenchymal stem cells**

**Hyun Jik Lee^1,^ *, Young Hyun Jung^1,^ *, Gee Euhn Choi^1^, Jun Sung Kim^1^, Chang Woo Chae^1^, Jae Ryong Lim^1^, Seo Yihl Kim^1^, Joo Eun Lee^1^, Min Chul Park^1^, Jee Hyeon Yoon^1^, Myeong Jun Choi^2^, Kye-Seong Kim^3^ and Ho Jae Han^1, #^**

**Affiliation**

^1^Department of Veterinary Physiology, College of Veterinary Medicine, Research Institute for Veterinary Science, and BK21 PLUS Program for Creative Veterinary Science Research, Seoul National University, Seoul 08826, Republic of Korea*.*

^2^Axcesobiopharma, 268, Hakuiro, Dongan-gu, Anyang 14056, Republic of Korea.

^3^Department of Biomedical Science, Graduate School of Biomedical Science and Engineering, Hanyang University, 222 Wangsimni-ro, Seongdong-gu, Seoul 04763, Republic of Korea.

*****These authors contributed equally to this work.

**^#^**Corresponing author: Ho Jae Han, D.V.M., Ph.D.

Department of Veterinary Physiology, College of Veterinary Medicine and Research Institute for Veterinary Science, Seoul National University, Seoul 08826, Republic of Korea

Tel: +82-2-880-1261

Fax: +82-2-880-2732

E-mail: hjhan@snu.ac.kr

Running Title: Regulatory effect of cP1P on mesenchymal stem cells


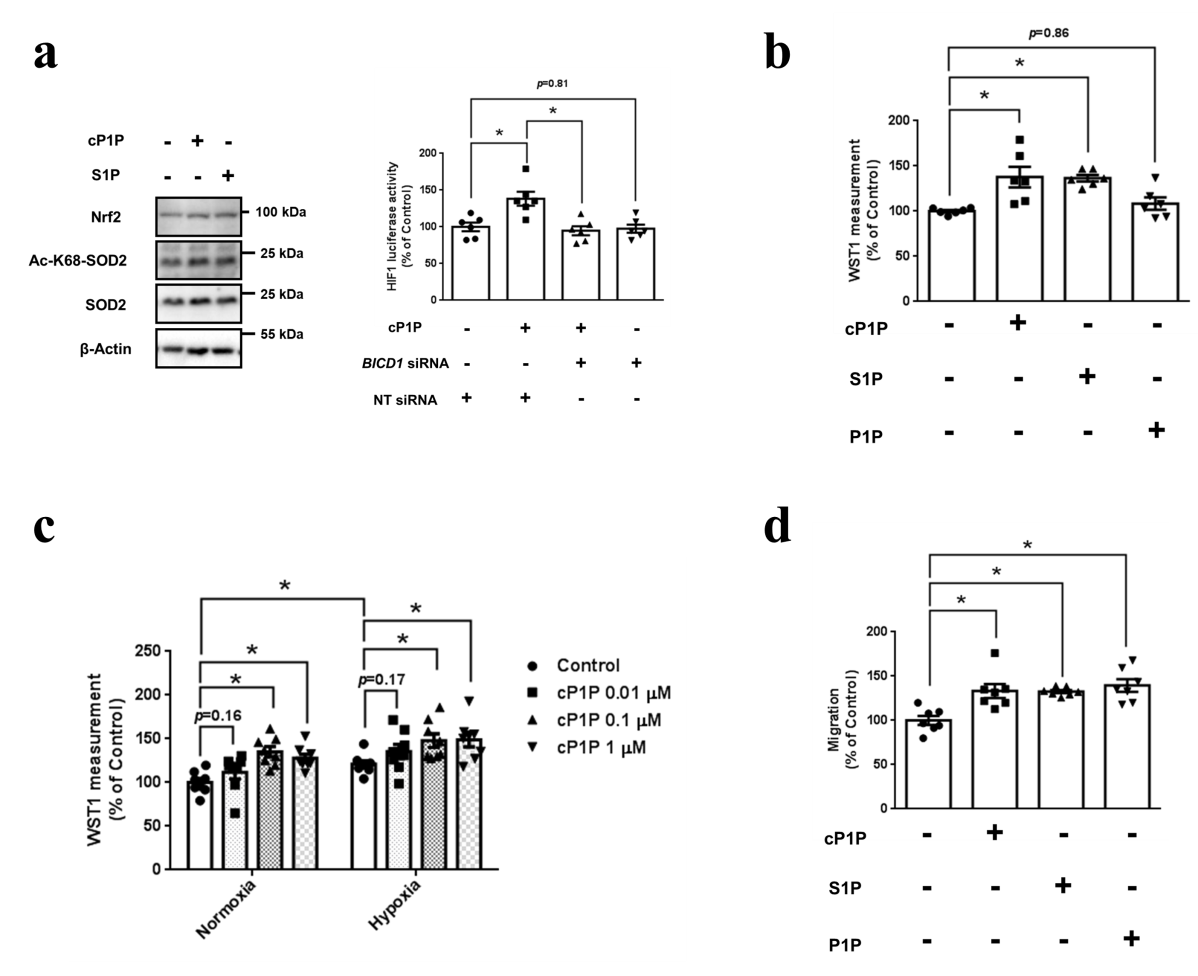


**Supplementary figure S1.** Effects of sphingosine metabolites on Nrf2 expressio, SOD2 acetylation, proliferation, and migration of UCB-MSCs. **(a)** UCB-MSCs were treated with cP1P (1 μM) or S1P (1 μM) for 24 h. Nrf2, Ac-K68-SOD2, SOD2, and β-Actin expressions were detected by western blotting. *n* = 4. **(b)** UCB-MSCs were treated with cP1P (1 μM), S1P (1 μM), or P1P (1 μM) for 48 h. Proliferation rates of UCB-MSCs were assessed by WST-1 cell proliferation assay. **(c)** UCB-MSCs were treated with cP1P (0.01, 0.1, or 1 μM) for 48 h under hypoxia. Proliferation rates of UCB-MSCs were assessed by WST-1 cell proliferation assay. **(d)** UCB-MSCs were treated with 1 μM of cP1P, S1P, or P1P for 24 h. Cell migration rates of UCB-MSCs were assessed by Oris migration assay. All blot images are representative. Quantitative data are presented as a mean ± S.E.M with scatter plots. * indicates *p* < 0.05.


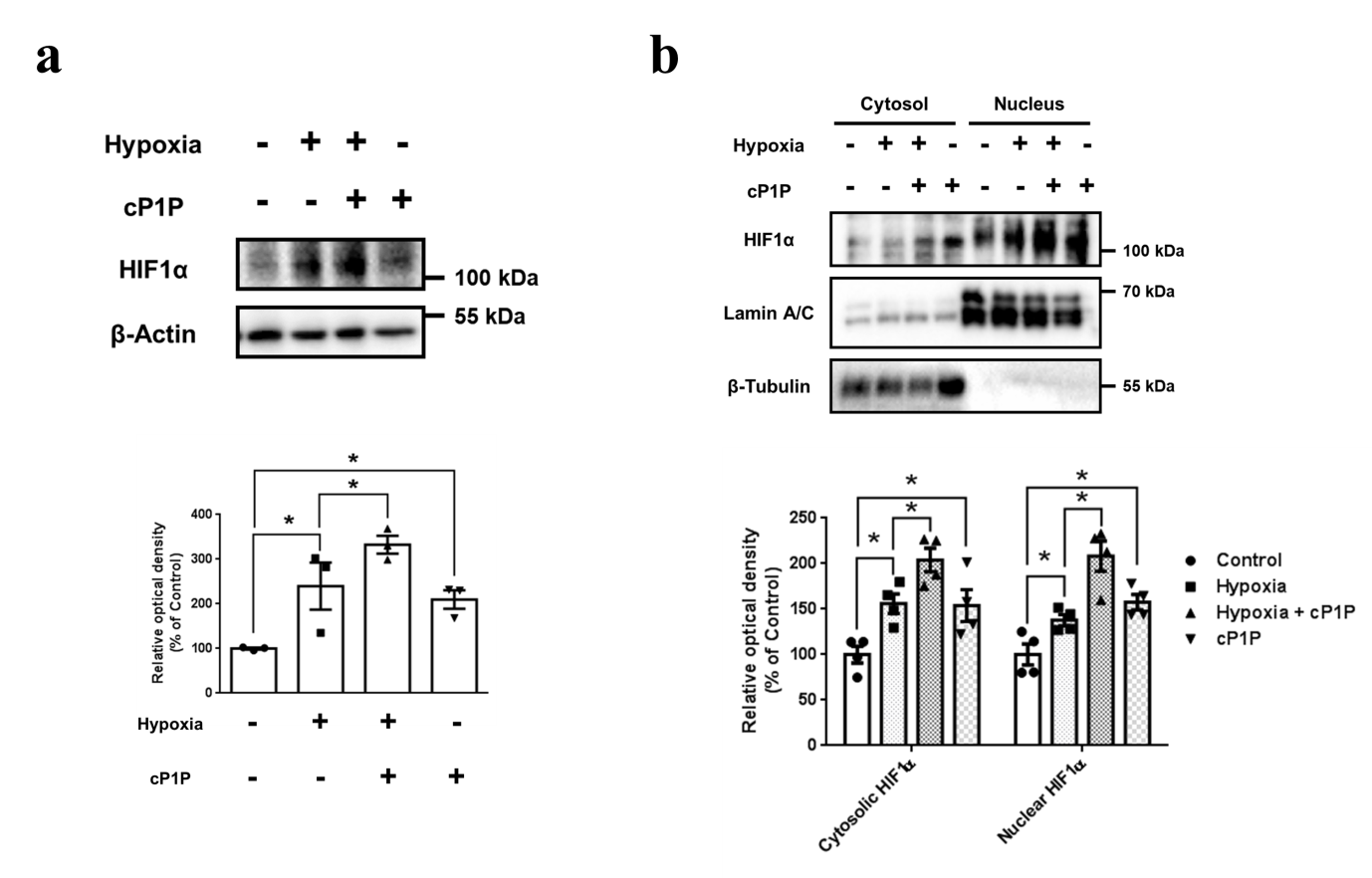


**Supplementary figure S2.** Effect of cP1P and hypoxia on HIF1α expression. **(a, b)** UCB-MSCs were pretreated with cP1P (1μM) for 30 min prior to hypoxia treatment for 48 h. **(a)** Total protein expressions of HIF1α and β-Actin were detected by western blotting. *n* = 3. **(b)** HIF1α, lamin A/C, and β-Tubulin protein expressions in cytosolic or nuclear fractionated samples were detected by western blotting. *n* = 4. All blot images are representative. Quantitative data are presented as a mean ± S.E.M with scatter plots. * indicates *p* < 0.05.


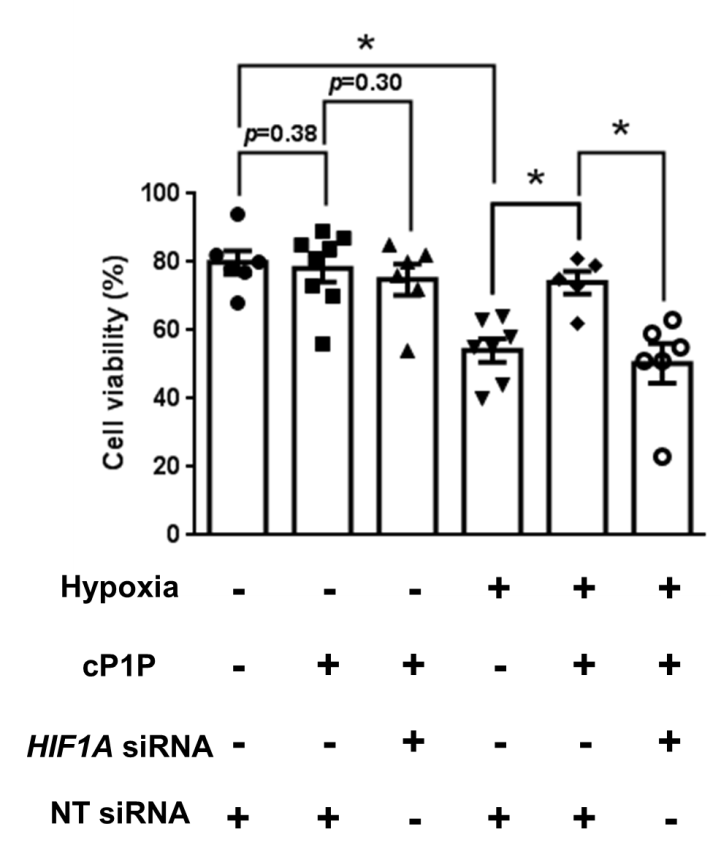


**Supplementary figure S3.** Effects of *HIF1A* silencing and cP1P on UCB-MSC survival under hypoxia. NT or *HIF1A* siRNA-transfected UCB-MSCs pretreated with cP1P (1 μM) for 30 min prior to hypoxia treatment for 72 h. The cell viabilities of UCB-MSCs under normoxia or hypoxia were measured by trypan blue exclusion cell viability assay. *n* = 6-8. Quantitative data are presented as a mean ± S.E.M with scatter plots. * indicates *p* < 0.05.


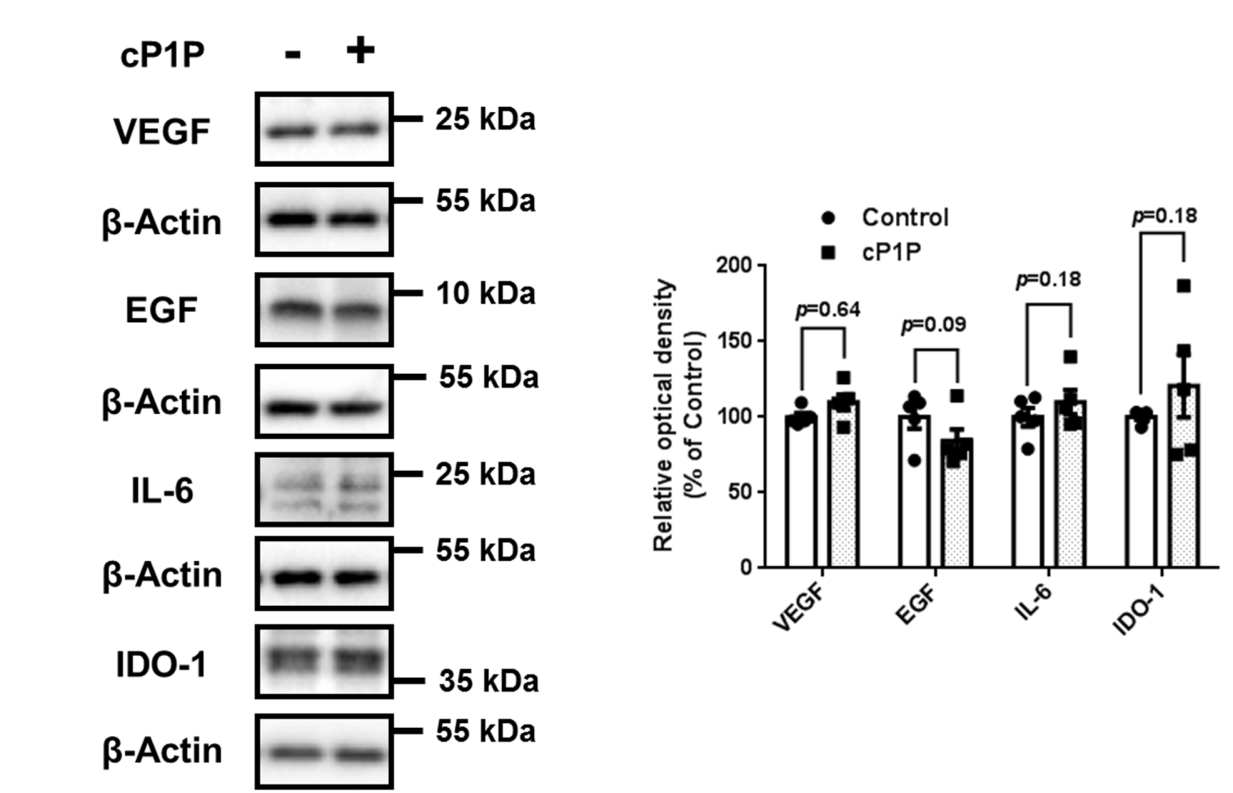


**Supplementary figure S4.** Effect of cP1P on VEGF, EGF, IL-6, and IDO-1 expressions in UCB-MSCs. UCB-MSCs were treated with cP1P (1 μM) for 24 h. The protein expressions of VEGF, EGF, IL-6, IDO-1, and β-Actin were detected by western blotting. *n* = 5. All blot images are representative. Quantitative data are presented as a mean ± S.E.M with scatter plots. * indicates *p* < 0.05.


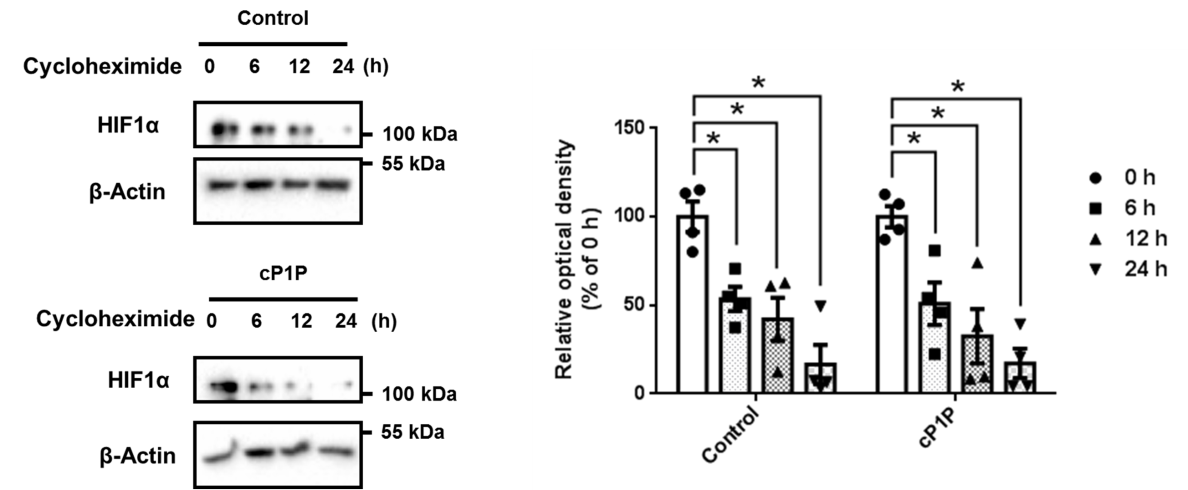


**Supplementary figure S5**. Effect of cP1P on HIF1α protein stability in UCB-MSCs. UCB-MSCs were pretreated with cP1P (1 μM) for 30 min. UCB-MSCs were then exposed to cycloheximide (20 μg/ml). Cells were collected at indicated time-points. The protein expressions of HIF1α and β-Actin were detected by western blotting. *n* = 4. All blot images are representative. Quantitative data are presented as a mean ± S.E.M with scatter plots. * indicates *p* < 0.05.


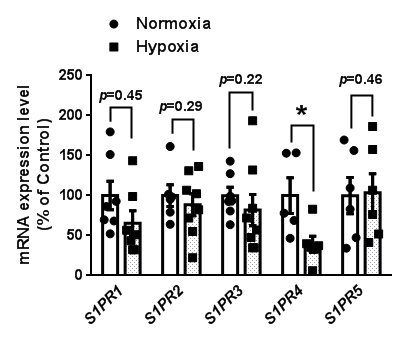


**Supplementary figure S6.** Effect of hypoxia on S1PRs mRNA expressions. UCB-MSCs were incubated in hypoxia for 24 h. The mRNA expressions of *S1PR1*, *S1PR2*, *S1PR3*, *S1PR4*, and *S1PR5* in UCB-MSCs were normalized by *18S rRNA* expression levels. *n* = 6-8. Quantitative data are presented as a mean ± S.E.M with scatter plots. * indicates *p* < 0.05.


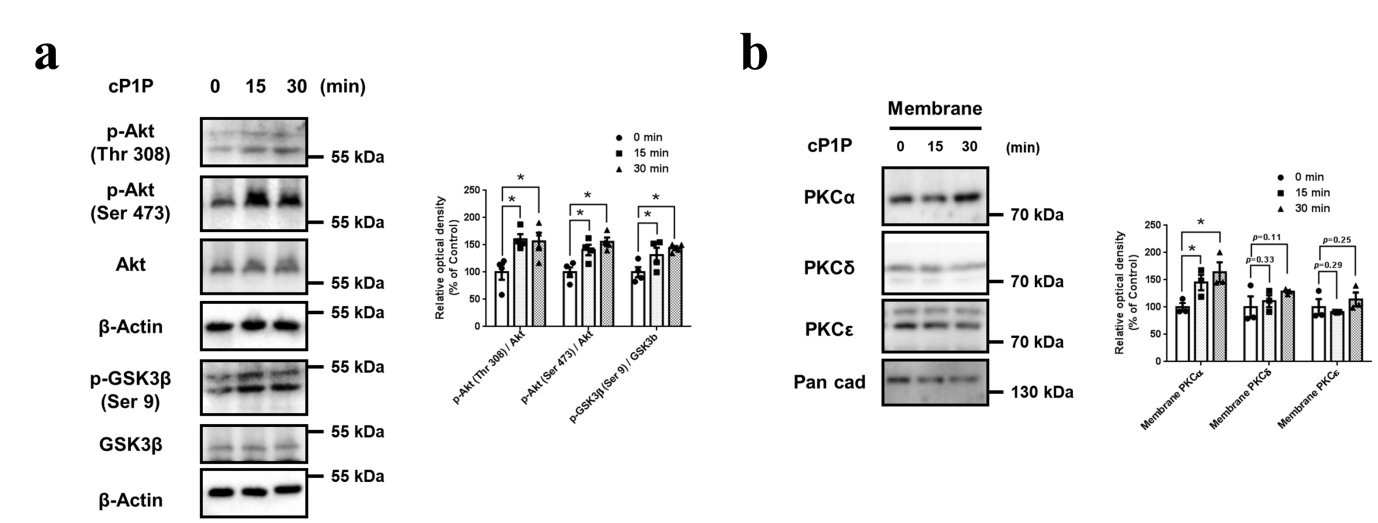


**Supplementary figure S7.** Effect of cP1P on Akt, GSK3β phosphorylations, and PKCs membrane translocation. **(a, b)** UCB-MSCs were treated with cP1P (1 μM) for 0, 15, or 30 min. **(a)** The protein expressions of p-Akt (Thr 308), p-Akt (Ser 473), Akt, p-GSK3β (Ser 9), GSK3β, and β-Actin were detected by western blotting. *n* = 4. **(b)** The protein expressions of PKCα, PKCδ, PKCε, and pan-cad in membrane fractionated samples were detected by western blotting. *n* = 3. All blot images are representative. Quantitative data are presented as a mean ± S.E.M with scatter plots. * indicates *p* < 0.05.


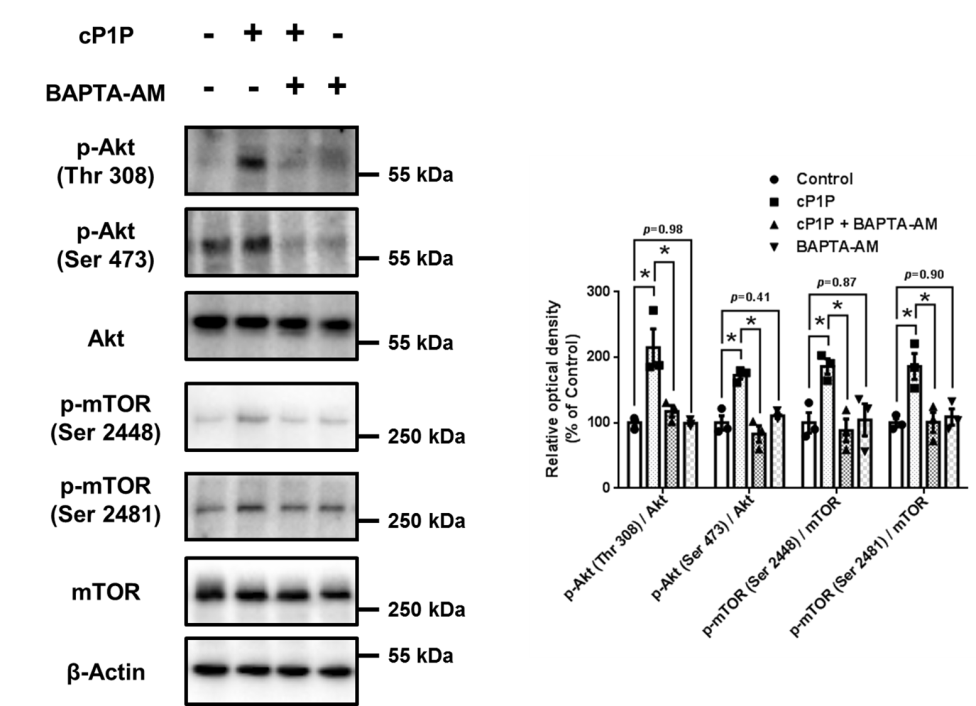


**Supplementary figure S8.** Role of calcium in cP1P-induced Akt/mTOR pathway. UCB-MSCs were pretreated with BAPTA-AM (10 μM) for 30 min prior to cP1P (1 μM) for 24 h. The protein expressions of p-Akt (Thr 308), p-Akt (Ser 473) Akt, p-mTOR (Ser 2448), p-mTOR (Ser 2481), mTOR, and β-Actin were detected by western blotting. *n* = 3. All blot images are representative. Quantitative data are presented as a mean ± S.E.M with scatter plots. * indicates *p* < 0.05.


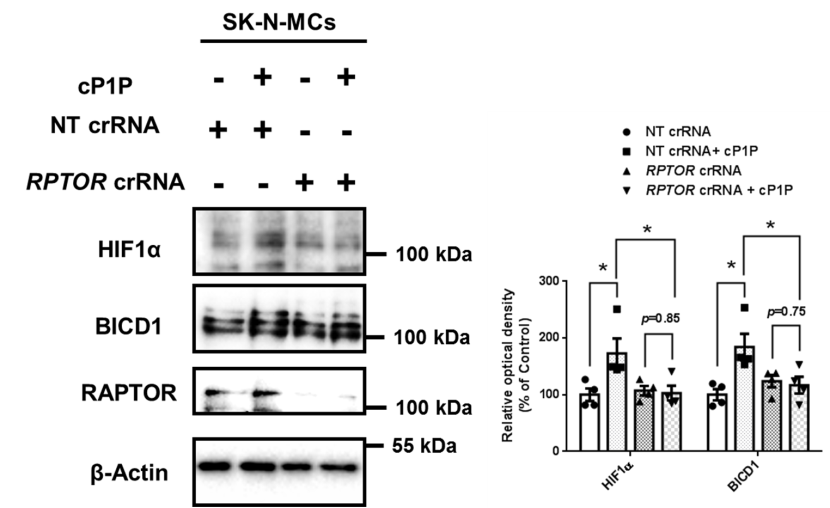


**Supplementary figure S9.** Effect of *RPTOR* gene knock out in cP1P-induced HIF1α and BICD1 expressions of SK-N-MCs. cP1P (1 μM) was treated to *RPTOR* gene knock out or control SK-N-MCs for 24 h. HIF1α, BICD1, and β-Actin were detected by western blotting. *n* = 4. All blot images are representative. Quantitative data are presented as a mean ± S.E.M with scatter plots. * indicates *p* < 0.05.

**Table S1. Sequences of primers used for RT-PCR and real-time PCR**

| Gene | Identification | Sequence (5'-3') | Size (bp) |
| --- | --- | --- | --- |
| *LDHA* | Sense | ACGTCAGCAAGAGGGAGAAA | 191 |
|  | Antisense | CGCTTCCAATAACACGGTTT |  |
| *PDK1* | Sense | CACGCTGGGTAATGAGGATT | 243 |
|  | Antisense | ACTGCATCTGTCCCGTAACC |  |
| *BICD1* | Sense | TCCATCCACCGGAAGGTTG | 127 |
|  | Antisense | GGCTCTGTTTCAGCTCGTTC |  |
| *HIF1A* | Sense | GTTTACTAAAGGACAAGTCACC | 193 |
|  | Antisense | TTCTGTTTGTTGAAGGGAG |  |
| *NHE1* | Sense | TCCATGCAGAACATCCACCC | 188 |
|  | Antisense | AGCATCTGGTTCCAGGCTTC |  |
| *S1PR1* | Sense | CGAGAGCACTACGCAGTCAG | 193 |
|  | Antisense | GAGAGCCTTCACTGGCTTCA |  |
| *S1PR2* | Sense | CGGCCTAGCCAGTTCTGAAA | 196 |
|  | Antisense | CAATGGCGCAACAGAGGATG |  |
| *S1PR3* | Sense | GCACGCCTTGCTGAATGAAG | 183 |
|  | Antisense | GTACTGGTAATGCTCCCGCA |  |
| *S1PR4* | Sense | AGTCTTGCGTGTGGATGGTG | 175 |
|  | Antisense | GACCATGGGAAGCCCATTTG |  |
| *S1PR5* | Sense | GACCCCTCCGAATCATCGAC | 154 |
|  | Antisense | GAGCTTGCCGGTGTAGTTGT |  |
| *18S rRNA* | Sense | GGCCGTTCTTAGTTGGTGGA | 183 |
|  | Antisense | CCCGGACATCTAAGGGCATC |  |

**Table S2. Sequences of siRNAs used for gene silencing**

| Target gene | Sequence | Supplier |
| --- | --- | --- |
| *SIPR1* | CUCUUCAGAGCGGAGUACU  AGUACUCCGCUCUGAAGAG | Bioneer |
|  | GAUAUCAUCGUCCGGCAUU  AAUGCCGGACGAUGAUAUC |  |
| *S1PR3* | GAGUCUUUCAGAUGUACUA  UAGUACAUCUGAAAGACUC | Bioneer |
|  | GUAGAAUGGAUGCUUGUAU  AUACAAGCAUCCAUUCUAC |  |
| *HIF1A* | GUGGUUGGAUCUAACACUA  UAGUGUUAGAUCCAACCAC | Bioneer |
|  | CAUGAAAGCACAGAUGAAU  AUUCAUCUGUGCUUUCAUG |  |
|  | CUCAUCCAUGUGACCAUGA  UCAUGGUCACAUGGAUGAG |  |
| *RPTOR* | CGAGAUUGGACGACCAAAUTT  AUUUGGUCGUCCAAUCUCGTT | Bioneer |
|  | GGUGAACUGAACUGGAUCUTT  AGAUCCAGUUCAGUUCACCTT |  |
|  | CCGAGAAUGAGGAGCAUAUTT  AUAUGCUCCUCAUUCUCGGTT |  |
|  | GCGUCACACUGGAUUUGAUTT  AUCAAAUCCAGUGUGACGCTT |  |
| *BICD1* | CUGAACACUUUGUUACGAA  UUCGUAACAAAGUGUUCAG | Bioneer |
|  | GAGGAUGGGAGUGAACCAA  UUGGUUCACUCCCAUCCUC |  |
|  | GCUAGGGAAGUUGAUUCCA  UGGAAUCAACUUCCCUAGC |  |
| Non-targeting (NT) | UAGCGACUAAACACAUCAA | Dharmacon |
|  | UAAGGCUAUGAAGAGAUAC |  |
|  | AUGUAUUGGCCUGUAUUAG |  |
|  | AUGAACGUGAAUUGCUCAA |  |

**Table S3. Scoring of histological changes in skin flap**

| **Score** | **Re-epithelialization** |
| --- | --- |
| **0** | Absence of epithelial proliferation in > 70 % of the tissue |
| **1** | Poor epidermal organization in > 60 % of the tissue |
| **2** | Incomplete epidermal organization in > 40 % of the tissue |
| **3** | Moderate epithelial proliferation in > 60 % of the tissue |
| **4** | Complete epidermal remodeling in > 80 % of the tissue |

**Table S4. List of upregulated glycolysis regulating genes by cP1P treatment**


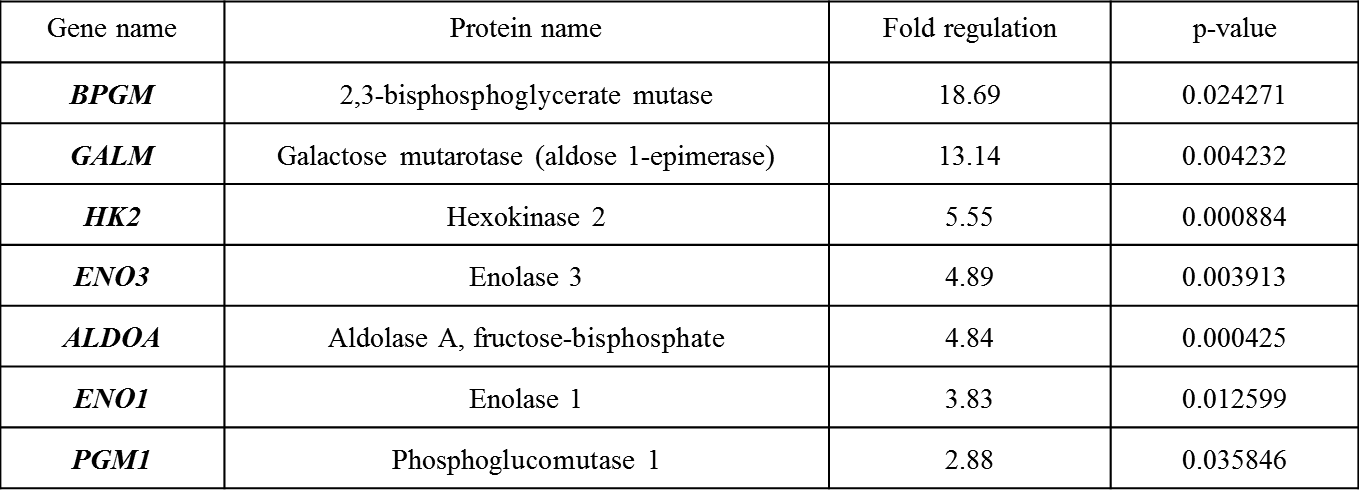


**Table S5. List of downregulated TCA cycle regulating genes by cP1P treatment**


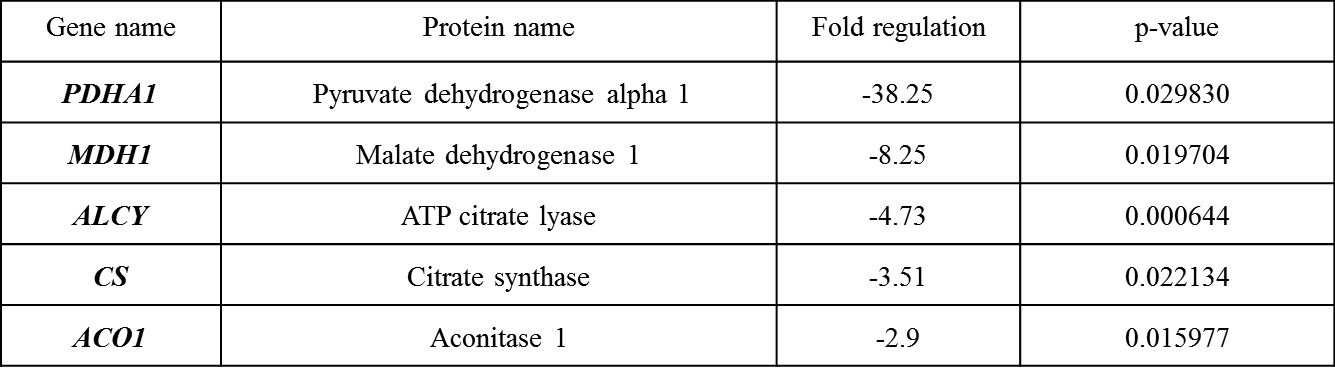

Supplement: Supplementary file 1 — Supplementary material. [file 41419_2019_1823_MOESM1_ESM.docx]
